# SMARThealth India: A stepped-wedge, cluster randomised controlled trial of a community health worker managed mobile health intervention for people assessed at high cardiovascular disease risk in rural India

**DOI:** 10.1371/journal.pone.0213708

**Published:** 2019-03-26

**Authors:** David Peiris, Devarsetty Praveen, Kishor Mogulluru, Mohammed Abdul Ameer, Arvind Raghu, Qiang Li, Stephane Heritier, Stephen MacMahon, Dorairaj Prabhakaran, Gari D. Clifford, Rohina Joshi, Pallab K. Maulik, Stephen Jan, Lionel Tarassenko, Anushka Patel

**Affiliations:** 1 The George Institute for Global Health, University of New South Wales, Sydney, Australia; 2 The George Institute for Global Health, Hyderabad, India; 3 Institute of Biomedical Engineering, Department of Engineering Science, University of Oxford, Oxford, United Kingdom; 4 School of Public Health and Preventive Medicine, Monash University, Melbourne, Australia; 5 The George Institute for Global Health, University of Oxford, Oxford, United Kingdom; 6 Centre for Chronic Disease Control, New Delhi, India; 7 London School of Hygiene and Tropical Medicine, London, United Kingdom; 8 Public Health Foundation of India, Delhi, India; 9 Department of Biomedical Informatics, Emory University, Atlanta, Georgia, United States of America; 10 Department of Biomedical Engineering, Georgia Institute of Technology, Atlanta, Georgia, United States of America; 11 The George Institute for Global Health, New Delhi, India; La Trobe University, AUSTRALIA

## Abstract

**Background:**

Cardiovascular diseases (CVD) are rising in India resulting in major health system challenges.

**Methods:**

Eighteen primary health centre (PHC) clusters in rural Andhra Pradesh were randomised over three, 6-month steps to an intervention comprising: (1) household CVD risk assessments by village-based community health workers (CHWs) using a mobile tablet device; (2) electronic referral and clinical decision support for PHC doctors; and (3) a tracking system for follow-up care. Independent data collectors screened people aged ≥ 40 years in 54 villages serviced by the PHCs to create a high CVD risk cohort (based on WHO risk charts and blood pressure thresholds). Randomly selected, independent samples, comprising 15% of this cohort, were reviewed at each 6-month step. The primary outcome was the proportion meeting systolic blood pressure (SBP) targets (<140mmHg).

**Findings:**

Eight-four percent of the eligible population (n = 62,254) were assessed at baseline (18.4% at high CVD risk). Of those at high risk, 75.3% were followed up over two years. CHWs screened 85.9% of the baseline cohort and doctors followed up 70.0% of all high risk referrals. There was no difference in the proportion of people achieving SBP targets (41.2% vs 39.2%; adjusted odds ratio (OR) 1.01 95% CI 0.76–1.35) or receiving BP-lowering medications in the intervention vs control periods respectively. There was a high discordance in risk scores generated by independent data collectors and CHWs, resulting in only 37.2% of the evaluation cohort exposed to the intervention. This discordance was mainly driven by fluctuating BP values (both normal variability and marked seasonal variations). In the pre-specified high risk concordant subgroup, there was greater use of BP-lowering medications in the intervention period (54.3% vs 47.9%, OR 1.22, 95% CI 1.03–1.44) but no impact on BP control.

**Conclusions:**

The strategy was well implemented with increased treatment rates among high risk individuals assessed by CHWs, however effects on BP were not demonstrated. Use of guideline-recommended BP thresholds for identifying high risk individuals substantially affected the reproducibility of risk assessment, and thus the ability to reliably evaluate the effectiveness of the intervention. In addition, unanticipated seasonal variation in BP in the context of a stepped-wedge trial highlights the inherent risks of this study design.

**Trial registration:**

Clinical Trials Registry of India CTRI/2013/06/ 003753.

## Introduction

Cardiovascular diseases (CVD) are a major cause of premature morbidity and mortality globally, with ischemic heart disease and stroke responsible for 24% of all deaths.[1] In India, although there are challenges with data quality, the number of years of life lost because of coronary heart disease deaths before the age of 60 years is projected to increase from 7·1 million in 2004 to 17·9 million in 2030, more life years lost than is projected for China, Russia, and the USA combined.[2] Elevated blood pressure (BP) is a major contributor to the increasing burden of CVD in India, causing almost a million deaths annually.[2] India has an estimated 140 million people diagnosed with hypertension with projections indicating an increase to 213 million by 2025.[3] In rural areas (defined by the Reserve Bank of India as tier-3 to tier-6 cites <50,000 people), where almost 70% of the country’s population resides, high levels of hypertension and other CVD risk factors exist and CVD is the leading cause of adult deaths.[4–6]

In 2008, the Indian central government launched the National Program for prevention and control of Cancer, Diabetes, Cardiovascular diseases, and Stroke (NPCDCS) with two key areas of focus: early detection of persons with high levels of risk factors and strengthening the health system to tackle non-communicable diseases (NCDs). [7] At the core of the guidelines used in this program is the assessment of absolute 10-year CVD risk using the World Health Organization/ International Society for Hypertension (WHO/ISH) risk charts and recommendation of medication to those identified with or at high risk of CVD. Despite a national policy being in place and mandated availability of low cost antihypertensive drugs in government formularies, the use of BP lowering treatments in rural India is limited, with few people with hypertension and/or CVD appropriately managed in these settings.[8, 9] Overall only 39% of all individuals either with previous CVD or at high risk of CVD have adequate BP control, indicating large evidence-practice gaps and ineffective current approaches to reducing BP-related risk.[8]

India’s health system faces great challenges to improve coverage and quality of care for its citizens. Reduced numbers of health care facilities and care providers, reliance on informal and private care providers, and high out-of-pocket costs are major barriers that need addressing.[10] One promising solution is to expand the capacity of community health workers (CHWs) with lower levels of health training by supporting them to share tasks traditionally performed by a doctor. [11, 12] Currently, rural primary health care is delivered at a Primary Health Centre (PHC) facility which is usually led by one doctor and services around 30,000 residents from the surrounding villages and nurse/ midwife level care is provided at sub-centres which support around 3000–5000 people. At the village level, for every 1000 population, one female resident is appointed as Accredited Social Health Activist (ASHA). ASHAs receive performance-based remuneration under India’s National Rural Health Mission programme. [13] On average, they work for 2–3 hours each day, with a primary focus on maternal and child health.[14] A cluster randomised controlled trial (c-RCT) of a cardiovascular risk screening strategy involving 44 villages in the region demonstrated that a paper-based algorithm administered in the community by CHWs increased the detection of CVD. [15],[16] This suggests that this workforce can be trained to effectively identify people at high risk for CVD and refer them appropriately for medical care. India is enacting policies to support the enhancement of task shifting models. The recently announced Ayushman Bharat National Health Protection Scheme plans to upgrade over 150,000 subcentres to health and wellness centres and a core element of these subcentres will be staffing by non-doctor, multipurpose workers.[17]

Mobile technologies have the potential to increase access to health care and health information on a large scale. Although there is potential for these devices to transform the delivery of health care, especially in low and middle-income countries (LMICs), the current evidence for their effectiveness is fragmented.[18] Further, most mHealth apps rely on siloed functions that are not integrated with existing provider systems and there are few robust implementation trials that have been conducted to assess the effectiveness and cost-effectiveness of these interventions. [19]

To address some of these limitations, we developed SMARThealth India—a multifaceted primary health care intervention to support PHC doctors and village-based ASHAs in the provision of guidelines-based assessment and management of CVD risk. [20, 21] We hypothesised that when compared to usual practice, a primary healthcare worker led clinical decision support system will increase the proportion of high risk individuals achieving guideline-recommended BP levels.

## Methods

### Study design and participants

The intervention was evaluated using a stepped-wedge cluster randomized, controlled trial (cRCT) of two years’ duration. The study protocol has previously been published elsewhere. [20] The intervention focussed on a primary health service strategy and consequently a cluster design was considered most appropriate. A stepped-wedge design was chosen for two main reasons: (1) community and stakeholder consultation recommended offering the intervention to all participating sites and (2) a phased introduction of the intervention would yield important information on future scale up considerations.

### PHC and village eligibility

Clusters were defined at the level of the PHC. To be eligible each PHC had to be within 40km from a major town, have at least one doctor regularly providing services, but with all doctors willing to participate in the study. Villages were eligible if the majority of its population accessed health care from their designated PHC, and the population of the village was not small (defined as < 1900) or very large (above 10,000)—(tier-5 and tier-6 cities using the Reserve Bank of India classification). Villages also needed at least one ASHA willing to participate in the study per 1000 population. In those villages which lacked a government-appointed ASHA, we recruited ASHAs using the same selection criteria as for government recruitment. These include being resident of the village being serviced, qualified to year 10 education level and selected by the local government leadership (the gram panchayat). Of the 224 total ASHAs required, 189 were already working in the villages at the start of the intervention and an additional 35 ASHAs were recruited for the study.

Of the 29 PHCs within 40km from a major town (Bhimavaram), 5 were excluded due to the absence of a doctor. A total of 256 villages were serviced by the remaining 24 PHCs. 113 of these villages were excluded based on the small or large population criteria. From the remaining 143 villages, 3 villages were randomly selected per PHC leading to a total of 72 villages from 24 PHCs. These were then stratified into three groups according to the total population size per PHC, and 6 PHCs randomly selected from each group. In total 18 PHCs (with 3 villages randomly selected per PHC) in West Godavari District, Andhra Pradesh participated. (S1 Fig).

### Participant eligibility

Participants were eligible to participate if they were aged ≥ 40 years, classified at high CVD risk and indicated for BP lowering medication based on WHO and NPCDCS guidelines. High CVD risk and recommended for BP medication was defined as the presence of any of the following: (1) a past history of CVD confirmed by a doctor; (2) an extreme BP elevation (SBP >160mmHg or DBP >100mmHg); (3) a 10-year CVD risk ≥ 30%; (4) a 10-year CVD risk of 20–29% and a SBP>140 mmHg. The 10-year CVD risk of fatal or non-fatal major CVD event (myocardial infarction or stroke) was estimated using algorithms based on the WHO/ISH risk charts tailored to the South-East Asian Region-D.[22] Although these charts have not been formally validated in India, they are the recommended risk prediction equation in the NPCDCS guidelines. The simplified risk charts, which do not rely on cholesterol information, were used since availability and costs of cholesterol testing in this region limit its use. Individuals who were not able to provide informed consent were deemed to be ineligible to participate.

### Randomisation and masking

Cluster randomisation occurred at the level of the PHC. Randomisation of all 18 sites was conducted prior to commencement of the intervention at any PHCs. Following an initial 6-month control phase, six PHCs were randomised to the intervention over three time intervals or ‘steps’ of 6 months’ duration (Table 1) Central computer-based blinded randomisation was conducted with allocation stratified by population size.

**Table 1:** Stepped wedge allocation sequence.

	Time			
Steps	Months 0–6	Months 7–12	Months 13–18	Months 19–24
**Group 1** **(6 PHCs/ 18 villages)**	CONTROL	INTERVENTION	INTERVENTION	INTERVENTION
**Group 2** **(6 PHCs/ 18 villages)**	CONTROL	CONTROL	INTERVENTION	INTERVENTION
**Group 3** **(6 PHCs/ 18 villages)**	CONTROL	CONTROL	CONTROL	INTERVENTION

### Procedures

#### Intervention components (Fig 1)

**Fig 1:**
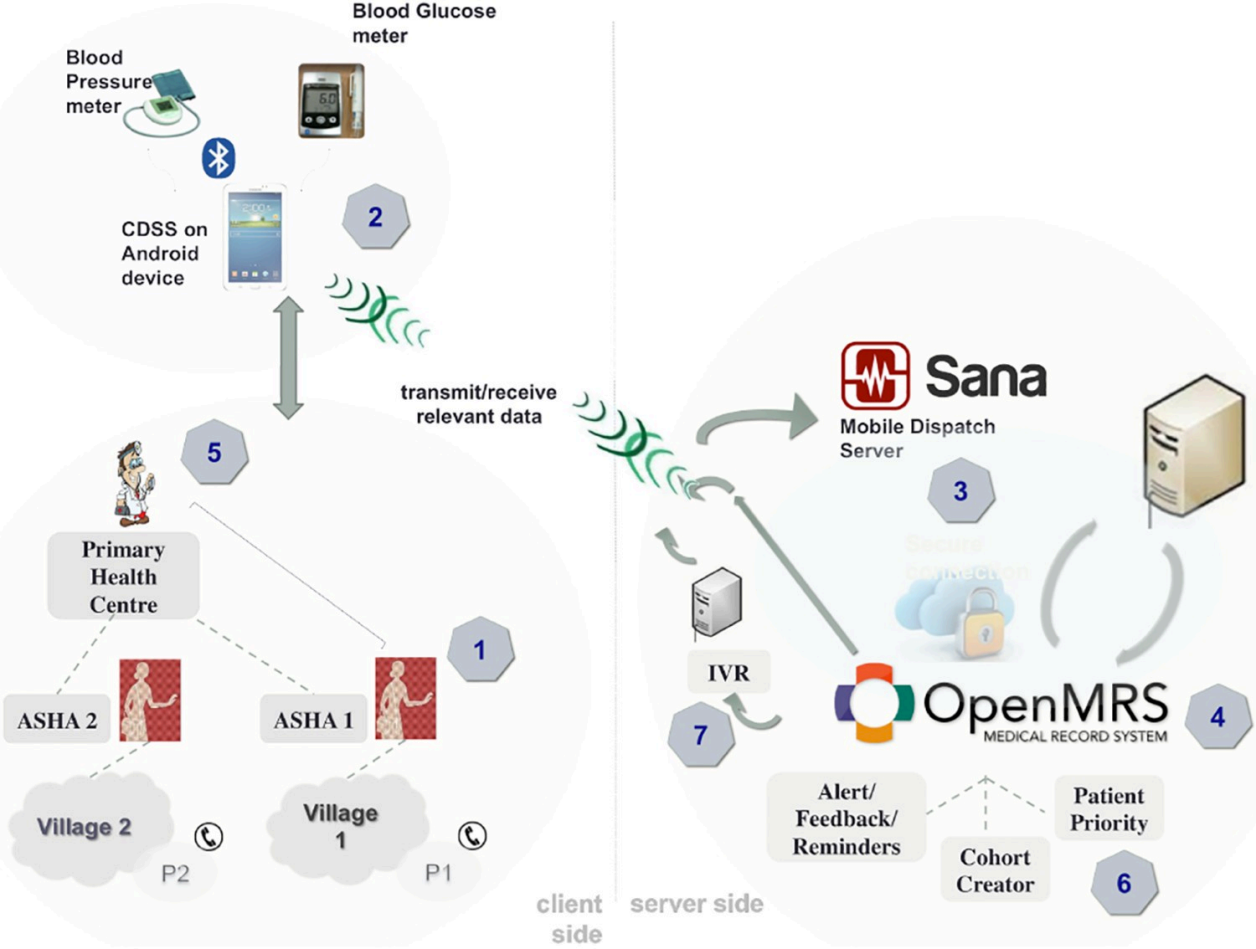
Intervention components. CDSS: Clinical Decision Support System; ASHA: Accredited Social Health Activist.

The intervention development and pilot testing has been described in detail elsewhere.[20, 23]

In brief, it comprised the following elements:

1. ASHAs and PHC doctors were trained to assess CVD risk using a local (Telugu) and English language clinical decision support system (CDSS) application on a 7-inch Android tablet device. The application allowed ASHAs to collect essential health-related information, inform the subject of their risk status, provide lifestyle advice relating to physical activity, diet and tobacco and alcohol, and refer high risk patients to the PHC doctor. In addition, the application provided decision support to doctors for medication prescription.
2. ASHAs received a 5-day training induction and ongoing support from field supervisors. Each ASHA was provided with a back pack sized kit, containing the tablet (Samsung Galaxy, model: T231), Bluetooth enabled BP monitor (A&D, model: UA-767PBT-C40, glucometer (Abbott, model: Freestyle Optimum) and other management resources (a hard copy training manual and a census list of each ASHA’s village population) were provided. Training was provided in selection of appropriate cuff size. Three BP readings were measured and the average of the last two readings were considered. Capillary glucose readings were measured. Participants were not required to be fasted for the glucose sample, and unless explicitly stated the sample was assumed to be random. Doctors received a one day induction and ongoing field support from a medically trained research fellow.
3. ASHAs conducted household-based assessments using the tablet device. Data were asynchronously uploaded to a shared electronic medical record (OpenMRS)[24] via the Sana Mobile Dispatch Server and stored on a centralised server. Internet connectivity (especially via 2G networks) is highly reliable in the study region and power outages range from 1 to 6 hours (worst in summer periods). ASHAs and PHC doctors did not have access to the data entered by the independent data collection team (described below) and conducted their assessments de novo.
4. Three modules were developed in OpenMRS to support patient tracking: (a) a cohort creator which facilitated grouping participants (e.g. those screened by an ASHA up to a particular point in time); (b) a patient priority module to help ASHAs prioritise workload for follow-up visits and screening of new participants; and (c) an alert module to provide feedback on whether patients were achieving recommended targets. OpenMRS can be integrated with government health information managements systems, however, this was not done in this study as such systems are seldom used in the PHC.
5. The doctors accessed the data uploaded by the ASHAs via OpenMRS and were provided with decision support recommendations for BP and other CVD risk factor management. Doctors were prompted to prescribe medications from the drug classes that were available on the essential medicine list in primary health care facilities and to enter a reason for not prescribing the medication if they considered it inappropriate.
6. Responses from the alert/reminder module (step 4) were used to create prompts in the ASHAs’ tablets to alert them to high risk individuals who required follow-up visits.
7. Patients received reminders on medication adherence and follow-up visits with the doctor via an interactive voice response system. This is an automated pre-recorded telephony service that notified patients via a one-way voice message of a particular action that was recommended to be taken.
8. A support team with five supervisors visited the ASHAs and doctors on a periodic basis and provided support for replacement of stocks (e.g glucose testing strips, batteries, swabs and faulty equipment), re-training, co-ordinating, and solving any IT issues. The quality of use of intervention was overseen by field supervisors who initially visited each ASHA two to three times per month for the first three months, reducing to monthly visits thereafter. Doctors and ASHAs were remunerated at comparable government rates for their time participating in the project. This included a sign-on payment for participating in the study and an incentive payment based on the number of patients assessed using the system. It amounted to an average of around 1500 INR per month per ASHA for 2 hours of work every day. This was in addition to payments received from the government for maternal and child health work.

### Control

During the control periods, participants in these PHCs/villages continued to receive their usual service provided by either a PHC doctor or a private doctor of their choosing. Any individuals identified at baseline to have extreme elevations of their BP were instructed to consult a doctor immediately.

### Data collection

#### Outcome dataset

Prior to randomisation, a baseline household survey of all consenting adults aged ≥ 40 years (average ~1000 households per village) was conducted in each village. An independent data collection team were trained to collect and enter information for CVD risk assessment into the 7-inch Android tablets in a manner identical to those used by the ASHAs in the intervention. This included identical protocols for BP measurement and data entry using Bluetooth enabled BP monitors. Those who were at high risk of CVD were identified, resulting in a census of all such individuals. Any individuals with extreme elevations of blood pressure or blood glucose were referred immediately to the PHC for treatment.

At each subsequent time point, follow-up data were collected on randomly selected, independent samples comprising 15% of the high risk cohort identified at baseline (average ~50 people per village/ 150 per PHC). This more conservative approach was adopted rather than following a baseline cohort in the expectation that the observed effects might be more replicable at scale. Individuals selected for follow-up were excluded from data collection at subsequent time-points to ensure independent samples at each time point. This approach to establish independent cross-sectional samples at each time point recognises that not all individuals identified at high risk at baseline will have a follow up visit for outcome evaluation. The data collection team were blinded to the allocation of the village. Outcome analyses were conducted with the statisticians blinded to intervention allocation.

#### Clinical dataset

In addition to the outcome dataset the clinical data collected by ASHAs and doctors as part of the intervention were also analysed for process measures to determine the result of the ASHA risk assessments, the proportions of patients followed up by doctors and ASHAs, the care provided by doctors and the proportions reported to be taking recommended medicines at follow-up visits by the ASHA.

### Outcomes

The outcome dataset was used to assess the trial outcomes. The primary outcome was the difference in proportion of high risk individuals achieving optimal BP levels (systolic BP<140 mmHg) between the intervention and control periods. Secondary outcomes included the difference in mean BP levels, difference in the proportion reporting use of BP medicines; difference in other CVD risk factors (body mass index; current smoking; self-reported dietary intake and physical activity levels); difference in quality of life (EQ-5D); and difference in the number of self-reported new CVD events.

Pre-specified patient level sub-groups included the following (1) attainment of the primary outcome at baseline; (2) BP treatment status at baseline; (3) past history of established CVD; (3) individuals identified by ASHAs to be at high CVD risk; (4) sex; and (5) use of a private vs. public doctor. Pre-specified PHC level sub-groups included: (1) PHC size based on attributed population; (2) PHCs with 80% or more government appointed ASHAs; and (3) availability of PHC doctors at the PHC for a minimum of 50% or more intervention time.

### Statistical analysis

Power was assessed by simulations for stepped-wedge trials with a uniform intra cluster correlation of 0.03 ((more conservative than the ICC of 0.01 previously observed in this population) and a binary outcome. We estimated that 18 PHC clusters of size 150 each, progressively randomised by a third to the intervention (Table 1) would provide >90% power (two-sided significance level of 5%) to detect an absolute difference of 6% in the proportion of people with optimal BP levels (defined as a systolic BP<140 mmHg). This translates to an increase in the proportion achieving optimal BP levels from 39% (based on previous data)[8] to 45% after small sample correction[25] and a mean systolic BP difference of around 3 mmHg. The calculation follows methods described by Hooper et al. accounting for repeated cross-sectional samples.[26]

Outcome analyses were conducted at the individual participant level. The primary analysis was originally based on mixed models as described by Hussey and Hughes.[27] As the primary endpoint is binary, a generalised linear mixed model (via SAS procedure GLIMMIX) was used with a logit link function, comparing the percentage achieving optimal BP levels between intervention and control periods with time as a fixed effect adjusted for baseline BP measurement and with clusters as a random effect. A correction to the robust variance estimate was also implemented to deal with the small number of clusters (18 PHCs).[28] Measures of association between the intervention and outcomes were presented as odds ratios (OR) with 95% confidence intervals and are conditional on the cluster effects of the logit link function. This correction is the default method for a small number of clusters in SAS. The same procedure was followed for categorical secondary outcome variables. Continuous secondary outcomes were modelled using linear mixed models with the same strategy and variance correction. Physical activity was defined as an ordinal endpoint with three categories (inactive, minimally active, active) and was analysed using a generalised-odds mixed model able that does not rely on the proportional-odds assumption. A secondary analysis was also pre-specified to adjust for any other potential imbalance factors at baseline. Generalised estimating equations (GEE) with a corrected sandwich formula were also conducted as part of a sensitivity analysis.

### Ethics

Ethical approval was granted by the Centre for Chronic Disease Control Institutional Ethics Committee and the University of Sydney Human Research Ethics Committee. The study was endorsed at the State level by the Government of Andhra Pradesh, at the district level by the West Godavari Director of Medical Services and at the village level by each Panchayat (the local governing council). Written informed consent was obtained from all participants contributing data prior to randomisation.

## Results

**Fig 2:**
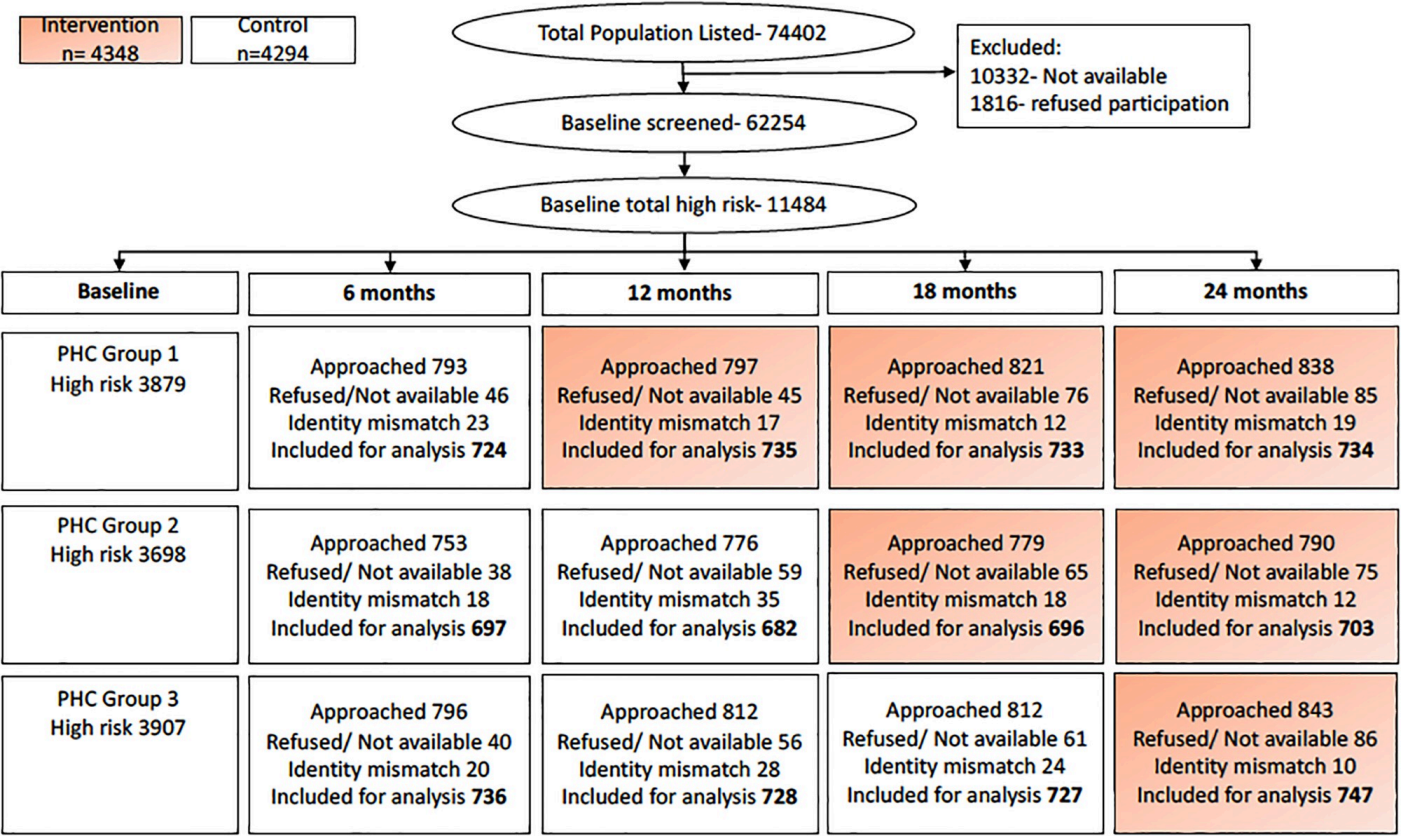
Study flow. PHC: Primary health centre.

Fig 2 outlines the study flow. Participants were recruited in June 2014 and follow-up data collection was completed in August 2016. In total 11,484 people were identified to be high risk at baseline and 8,642 of these (75.3%) were followed up over the four subsequent data collection time points with an average cluster size of 120 per PHC included in the analysis. This included 4294 who were followed up in the control period and 4348 who were followed up in the intervention period. Table 2 outlines the baseline characteristics for these two groups. Overall there were few differences in the two samples.

**Table 2:** Baseline characteristics for patients followed up in the control and intervention periods*.

	Control (n = 4294)	Intervention (n = 4348)
Age (years) mean (SD)	61.0 (10.86)	60.3 (10.71)
Male	1892 (44.1%)	1936 (44.5%)
Proportion never attended school	2013 (46.9%)	2034 (46.8%)
Occupation		
• Agricultural labourer	1007 (23.5%)	1045 (24.0%)
• Housewife	1371 (31.9%)	1341 (30.8%)
• Retired	990 (23.1%)	997 (22.9%)
• Other	251 (5.8%)	244 (5.6%)
Past history		
• Heart attack or angina	507 (11.8%)	515 (11.8%)
• Stroke	349 (8.1%)	399 (9.2%)
• Diabetes	941 (21.9%)	896 (20.6%)
• Hypertension	1997 (46.5%)	1986 (45.7%)
• Peripheral vascular disease	46 (1.1%)	23 (0.5%)
10 year CVD risk		
• 20—<30%	691 (16.1%)	696 (16.0%)
• 30—<40%	276 (6.4%)	262 (6.0%)
• > = 40%	215 (5.0%)	168 (3.9%)
• SBP > 160 mmHg or DBP > 100mmHg	2251 (52.4%)	2324 (53.4%)
• CVD	710 (16.5%)	755 (17.4%)
Current tobacco use	1099 (25.6%)	1155 (26.6%)
Physical inactivity	1444 (33.6%)	1404 (32.3%)
Self-reported current medication use		
• BP lowering	1805 (42.3%)	1810 (41.8%)
• Lipid lowering	159 (3.7%)	183 (4.2%)
• Anti-platelet	102 (2.4%)	116 (2.7%)
SBP less than 140mmHg	758 (17.7%)	791 (18.2%)
BMI (kg/m2)Mean (SD)	24.6 (5.05)	24.33 (4.98)
SBP (mmHg) Mean (SD)	157.3(23.23)	156.55 (22.90)
DBP (mmHg)Mean (SD)	88.8 (13.91)	89.11 (13.80)
Glucose (mg/dL) Mean (SD)	165.0 (81.14)	162.01 (81.60)
EQ5D utility score mean (SD)	0.800 (0.2166)	0.805 (0.2107)

*All proportions presented are prevalence rates

CVD: Cardiovascular diseases; SBP: Systolic Blood Pressure; DBP: Diastolic Blood Pressure; BMI: Body Mass Index; EQ5D: EuroQol 5-dimension questionnaire

**Fig 3:**
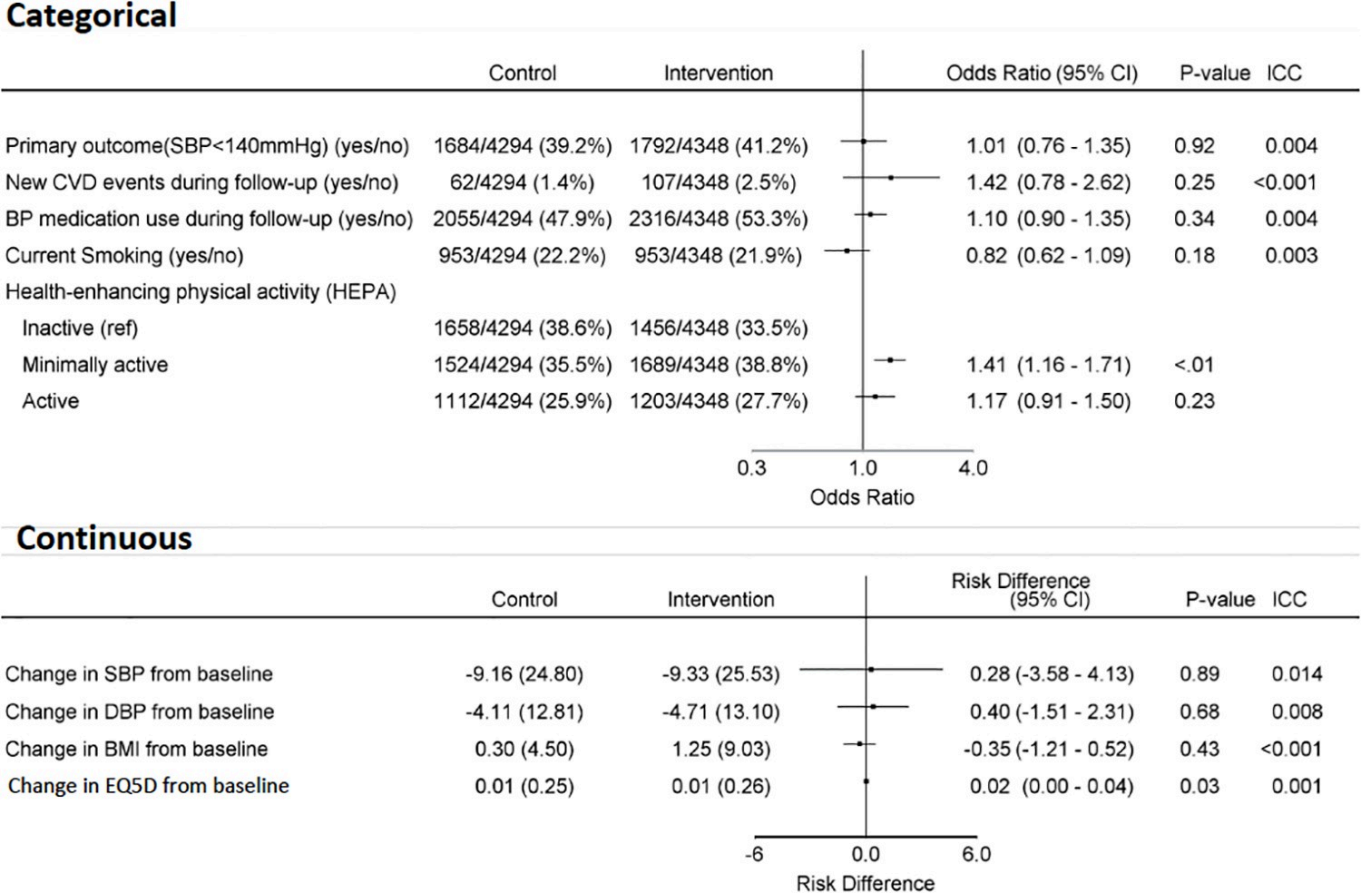
Primary and secondary outcomes by randomization period. SBP: systolic blood pressure; CVD: cardiovascular disease; DBP: diastolic blood pressure; BMI: body mass index; EQ5D: EuroQol quality of life instrument; ICC: intraclass correlation coefficient.

Fig 3 shows the primary and secondary outcomes by randomisation period. Overall there were no significant differences in any of the primary or secondary outcomes, aside from a small increase in self-reported physical activity. The proportion of people at high risk who were achieving the recommended BP target of SBP <140mmHg 41.2% vs 39.2% (OR_adj_ 1.01, 95% CI 0.76–1.35, ICC 0.004) in the intervention vs control periods respectively. The change in SBP from baseline was -9.3mmHg vs -9.2mmHg (mean difference 0.28, 95% CI -3.58–4.13, ICC 0.014) in the intervention vs control periods respectively. The proportion of people who reported taking at least one BP medication was 53.3% vs 47.9% (OR_adj_ 1.1, 95% CI 0.90–1.35, ICC 0.004) in the intervention vs control periods respectively. These findings did not substantively change with the pre-specified secondary analysis approaches, including adjustment for the following baseline covariates (age, sex, systolic blood pressure, body mass index, physical activity, smoking status and possession of a unique government identify number).

### Intervention fidelity

All available ASHAs in the 54 villages were recruited for the study (240 ASHAs). These ASHAs and 18 PHC doctors were trained in the use of the intervention package. ASHAs screened a total of 53,478 people (85.9% of the baseline cohort) and identified 8,419 (15.7%) to be at high CVD risk. Of these high risk individuals, the PHC doctor conducted at least one assessment for 70.0% and ASHAs conducted at least one follow-up visit for 85%. Follow-up was broadly uniform across all PHCs. Table 3 shows the degree of concordance in risk calculation between the baseline household data collectors and those assessed by ASHAs. The ASHAs identified only 4,280 of the 11,484 identified at high risk by the independent data collectors. Conversely a further 4,139 were identified by ASHAs at high risk who were assessed to not be at high risk by the independent data collectors.

**Table 3:** Concordance of CVD risk assessment between baseline data collectors and ASHA assessment.

	Baseline household census		
		Low/ mod CVD risk	High CVD risk
**ASHA risk assessment**	Low/ mod CVD risk	39432	5371
	High CVD risk	4139	4280
	Not screened	7082	1833
**Total**		**50653**	**11484**

The main driver for the high discordance rate was related to differences in recorded BP. A high proportion (53.8%) of the baseline high risk cohort of 11,484 people qualified as high risk solely on the basis of an elevated BP which was measured by the independent data team. At ASHA assessment, however, a large proportion of these individuals (45.6%) had subsequent BPs that placed them below the high risk threshold. Conversely others who were assessed at baseline as low risk had elevated BPs at ASHA assessment that newly put them at high CVD risk (47.3%).

In the pre-specified sub-group pertaining to the individuals identified at high risk by ASHAs who were exposed to the intervention there was no difference in the primary outcome (42.1% intervention vs 39.0% control, OR _adj_ 1.18, 95% confidence interval 0.97–1.44, p = 0.10, ICC <0.01). However, there was a significant improvement in use of BP medications (54.3% intervention vs 47.9% control, OR _adj_ 1.22, 95% confidence interval 1.03–1.44, p = 0.02, ICC <0.01). There were no other differences in outcomes for any of the other pre-specified sub-groups.

## Discussion

This study was a large scale trial of a complex intervention involving task-sharing between doctors and village-based community health workers using mHealth decision support. It was not effective in improving BP control rates for people at high CVD risk. Given the mHealth literature is dominated by small studies that are often not rigorously conducted, the findings from this trial are important. Despite great promise for mHealth interventions to improve access to effective health care, there remains considerable uncertainty about how this can be successfully achieved. These uncertainties pose substantial dilemmas for health system planners, particularly in LMICs, who are looking for affordable strategies to improve access to high quality health care in underserved regions.

A major strength of this study was the involvement of the vast majority of eligible individuals in 54 rural villages of a south Indian state, thereby enhancing generalizability of the findings to this region and other regions in India which have similar PHC infrastructures. The intervention was effectively integrated within routine health care service provision at the PHCs. It clearly demonstrates the potential to leverage the ASHA workforce across India and to expand their role beyond maternal and child health role to non-communicable disease management and prevention. The mHealth platform was highly effective in linking village based assessments to doctor level care and supported systematic follow-up care in the majority of patients who needed such care. The platform is highly scalable and amenable to use with different languages. Doctor engagement also was higher than expected with over 70% of ASHA-assessed high risk individuals followed up by the doctors. Given the workforce shortages of doctors it is a promising strategy to better utilize the medical workforce by engaging ASHAs to triage high risk individuals to the doctor. It also appears that the decision support provided to PHC doctors was effective as medication prescription was recommended to be newly initiated or continued in the majority of patients they reviewed (89%). Despite the high degree of doctor and ASHA engagement it is quite possible that there were several patient level barriers that minimized the impact of the intervention. At the first ASHA follow-up visit 25% reported not taking any medications despite being recommended these. Potential factors include both unintentional barriers to taking medicines (cost, transport, medication availability) and intentional non-adherence to recommended treatments.

There are three major limitations that might have contributed to the null outcome in this trial and indicate that further evaluation of effectiveness of the intervention is warranted. First and most important was the high discordance rate between the people identified at high CVD risk in the evaluation dataset and those identified by ASHAs. The major driver for this was related to the stepped wedge design and normal BP variability. ASHAs assessed people from 6 to 18 months after the baseline CVD risk assessment was conducted by the evaluation team. Consequently, only around 50% of the outcome evaluation cohort were exposed to the intervention and many people for whom outcome data were not collected, were exposed to the intervention. Given consistency of these findings across all ASHAs and the use of Bluetooth-enabled automatic BP machines, this discordance is unlikely to be driven by systematic measurement error by a selected number of ASHAs or the group as a whole. Rather, this raises the important limitations of using a high BP measurement taken at a single time point and using an arbitrary threshold to define individuals for whom BP lowering drugs would be appropriate. More than one clinical assessment may be required to better identify high risk individuals which has resource and guideline implications.

A second issue relates to seasonal fluctuation in BP levels and the impact this may have had with a stepped wedge design. There was a strong heatwave in Andhra Pradesh with temperatures as high as 48 degrees Celsius responsible for around 2000 deaths in the state in the second step of the trial, during which two thirds of the cohort were in the control period and one third in intervention. [29] There is a well-established inverse relationship between ambient temperatures and BP levels with one large study reporting a mean 5.7 mmHg SBP fall per 10 degrees Celsius higher temperature. [30] In the first 6 months when all PHCs were in the control period there was a mean 5.4 mmHg reduction in BP compared to baseline, which most likely represents regression to the mean. Following this, however, both intervention and control participants in the 2015 heatwave period exhibited much larger than expected reductions in mean SBP compared to baseline (-14.60mmHg reduction overall and -13.68mmHg reduction in untreated individuals). Given two thirds of the evaluation cohort were in the control period during the heatwave, this potentially biased our outcomes toward the null (S2 Fig). A parallel arm cRCT design would have mitigated this issue to some extent as any biases would have been evenly distributed in both trial arms. Temperature and humidity were significantly associated with the primary outcome and mean SBP and DBP changes. After adjustment for these variables in the ASHA-defined high risk sub-group, the odds ratio for the primary outcome favoured the intervention but remained non-significant (OR 1.20 (95% CI 0.85–1.70).

A third issue is related to a higher than expected improvement in BP treatment rates in the control period (a 5.6% absolute improvement). This could be related to known phenomena of control groups experiencing improvements when participating in trials but may also be due to background changes to usual practice. During the period of the trial the government of Andhra Pradesh strengthened the ‘104 service’ which is a private-public partnership providing a mobile health service to rural villages which includes chronic disease care, maternal and child health services and free access to essential medicines.[31] This program has proved to be highly popular in several villages and may be having a substantive impact on improving access to recommended treatments.

To obtain a more nuanced understanding of additional factors that might have impacted on the outcomes, a detailed process evaluation is currently underway which involves analyses of usage analytics extracted from the tablet devices, workforce capacity assessments before and after training modules were delivered, satisfaction surveys and in depth, semi-structured interviews involving ASHAs, doctors and participants. It is expected that these data will yield rich insights into contextual influences, potential mechanisms for how these interacted with the intervention (and which elements of the intervention) and the resulting outcomes.

Despite the outcomes in this trial, there are additional avenues for exploration to better understand the potential for mHealth and task sharing interventions in LMICs. These relate to (1) trialing different task sharing models, which may include an increased prescribing role for CHWs (currently not authorised in India), engagement of other workers such as nurses, pharmacists and allied health professionals; (2) increasing patient engagement strategies through use of mobile technology, cost subsidies for medicines and addressing unintentional adherence barriers; (3) assessing the intervention package in a different service delivery environment through private sector engagement; and (4) deeper integration of the intervention into the prevailing system incorporating factors such as supply chain management and human resource intervention. Assessment of such interventions, tailored to the environments in which they are delivered, will consider a wide set of levers for promoting evidence based treatment for CVD thereby substantially enriching the currently limited evidence base.

Although the strategy tested in this trial achieved a high uptake by doctors and community health workers, there were no statistically significant differences in outcomes when compared to usual care. Given the plethora of interventions worldwide involving mHealth and community health workers, this study provides useful information for those developing and testing similar strategies. For guideline developers there are also important insights into the way in which people at high risk of cardiovascular disease are defined, and designing the pathways for initiating treatment particularly given the large variations in BP measures observed in the same individuals. This is critically important for WHO guidelines given these guidelines greatly influence national NCD policies in many countries. For methodologists there are also important insights into the strengths and limitations of stepped wedge designs where seasonal trends in outcomes are pronounced. Improvements in these areas could greatly enhance the knowledge base on how to develop and rigorously evaluate multi-faceted strategies to improve NCD care in LMICs.

## Supporting information

S1 Fig Site selection. (TIFF)

S2 Fig Seasonal blood pressure variation (among subjects reporting no BP medication use at each visit). (TIFF)

S1 File Study protocol. (PDF)

S2 File Statistical analysis plan. (PDF)

S3 File Consolidated standards of reporting trials statement. (PDF)
