# Supplementary figures and images for: SMARThealth India: A stepped-wedge, cluster randomised controlled trial of a community health worker managed mobile health intervention for people assessed at high cardiovascular disease risk in rural India
Source: PLoS One. 2019 Mar 26;14(3):e0213708. doi: 10.1371/journal.pone.0213708 (PMC6435227; doi:10.1371/journal.pone.0213708)

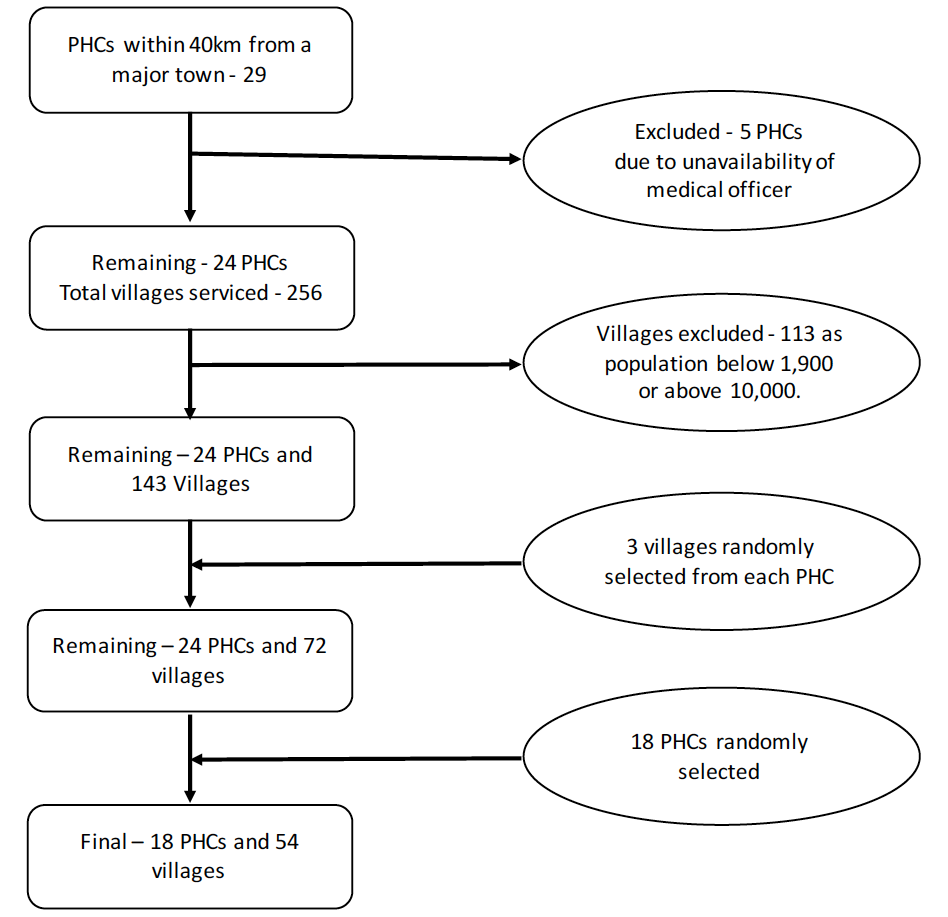

Supplement: S1 Fig — (TIFF) [file pone.0213708.s001.tiff]

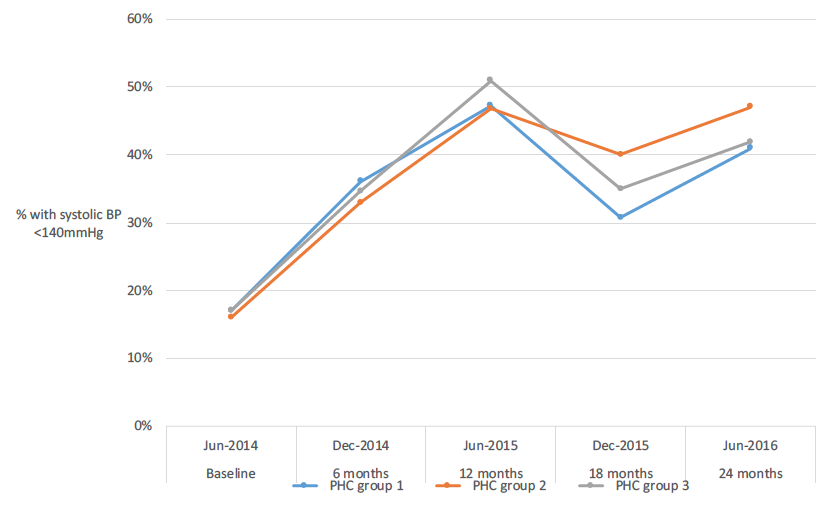

Supplement: S2 Fig — (TIFF) [file pone.0213708.s002.tiff]
