# Supplementary material for: SMARThealth India: A stepped-wedge, cluster randomised controlled trial of a community health worker managed mobile health intervention for people assessed at high cardiovascular disease risk in rural India
Source: PLoS One. 2019 Mar 26;14(3):e0213708. doi: 10.1371/journal.pone.0213708 (PMC6435227; doi:10.1371/journal.pone.0213708)
Supplement: S1 File — (PDF) [file pone.0213708.s003.pdf]

SMART Health- India

Systematic **M**edical **A**ssessment **R**eferral and **T**reatment in rural India

A stepped-wedge cluster randomised controlled trial of a primary health care mobile health system for cardiovascular risk management in rural Andhra Pradesh

STUDY PROTOCOL

Version 3, 12 September 2013

Contact Details:
The George Institute for Global Health
839C, Road No. 44A, Jubilee Hills
Hyderabad 500033, India
Tel +9140 2355 8091
Fax: +91 40 2354 1980
Email - info@georgeinstitute.org.in

TABLE OF CONTENTS

1 GLOSSARY OF TERMS 5

2 EXECUTIVE SUMMARY 6

3 STUDY SCHEMA 8

4 BACKGROUND 9

4.1 CVD burden in India 9

4.2 Evidence-practice gaps in CVD prevention 9

4.3 Workforce challenges 9

4.4 Clinical Decision Support Systems (CDSS) 10

4.5 Mobile-health interventions 10

5 SMARTHEALTH DEVELOPMENT 10

5.1 Decision support algorithm development 10

5.2 Shared electronic health record 11

6 STUDY OBJECTIVES 11

7 STUDY DESIGN 11

7.1 Study population 11

7.2 Randomisation 12

7.3 Intervention arm 12

7.4 Control arm 13

7.5 Data collection 13

7.6 Primary Outcomes 14

7.7 Secondary Outcomes 14

7.8 Statistical Considerations 14

7.9 Economic evaluation 14

7.10 Process evaluation 15

8 ETHICAL CONSIDERATIONS 15

9 TIMELINES 15

10 SIGNIFICANCE 16

11 ADMINISTRATIVE DETAILS 16

11.1 Steering Committee 16

11.2 Trial registration 16

11.3 Study monitoring 16

11.4 Protocol adherence 16

11.5 Protocol amendments 16

11.6 Study termination 17

11.6.1 Termination by the Steering Committee 17

11.6.2 Termination by the Investigator 17

11.6.3 Notification of Study Closure 17

11.7 Records retention 17

11.8 Indemnity 17

11.9 Publication and presentations 18

11.10 Funding 18

12 REFERENCES 19

13 APPENDIX 21

Steering Committee Membership 21

ABSTRACT

Backrgound: Hypertension related disease affected 118 million people in India in the year 2000; this figure will double by 2025. Our studies in rural India have found that 1 in 4 adults have hypertension and a minority are achieving adequate blood pressure (BP) control. The current health system infrastructure faces great challenges to meet these gaps in care and innovative solutions are needed.

Methods: In this study, we aim to bridge the implementation gap in BP control for individuals at high cardiovascular risk. We will assess the effectiveness of an innovative, multifaceted intervention that draws on three elements: (1) capacity strengthening of primary care doctors and non-physician health workers; (2) development of a mobile device-based clinical decision support system for use by these healthcare providers; and (3) integration of this system within the existing public primary healthcare sector.

Study Design and population: This intervention will be implemented as a stepped wedge cluster randomised controlled trial in 18 Primary Health Centres and 54 villages in rural Andhra Pradesh involving adults aged ≥40 years at high CVD risk (~15,000 people)

Expected Outcomes: We will assess whether the intervention improves control of BP and other cardiovascular risk factors when compared with usual health care. The trial outcomes will be accompanied by a detailed process and economic evaluation.

Signficance: The findings are likely to inform policy on a scalable strategy to overcome entrenched inequities in access to effective health care for under-served populations in low and middle income country settings.

1 GLOSSARY OF TERMS

ASHA	Accredited Social Health Activist
BP	Blood Pressure
CDSS	Clinical Decision Support System
cRCT	Cluster randomized controlled trial
CVD	Cardiovascular Disease
EQ-5D	EuroQuol-5D
HDL	High Density Lipoprotein
HREC	Human Research Ethics Committee
ICC	Intra-class correlation coefficient
IEC	Institutional Ethics Committee
ISH	International Society of Hypertension
LMIC	Low and Middle Income Countries
NPCDCS	National Programme for Prevention and Control of Cancer, Diabetes Cardiovascular and Stroke
PHC	Primary Health Centre
SMART	Systematic Medical Assessment Referral and Treatment
UAT	User Acceptance Testing
WHO	World Health Organisation

2 EXECUTIVE SUMMARY

Background
Diseases related to elevated blood pressure (BP) affected 118 million people in India in the year 2000; this figure will double by 2025. Our studies in rural India have found that 1 in 4 adults have elevated blood pressure and a minority are achieving adequate control. The current health system infrastructure is grossly under-resourced to meet these gaps in care and innovative solutions are needed. In this study, we aim to bridge the implementation gap in BP control for individuals at high cardiovascular risk.

Aims
The overall objective of this research is to investigate the effectiveness of an innovative and multi-disciplinary program addressing BP control in rural India. The specific aims are:

1. To develop a multifaceted primary healthcare worker (ASHA) intervention that utilises a mobile device-based clinical decision support system (CDSS) to improve optimal BP control in high risk individuals.
2. To evaluate this program utilising a mixed methods evaluation in a cluster randomised trial involving villages in rural Andhra Pradesh.

Study design
Stepped-wedge, cluster, randomized controlled trial

Study population
Adults ≥40 years at high cardiovascular disease (CVD) risk, defined as either (1) established CVD (either coronary heart disease, stroke/ transient ischemic attack or peripheral vascular disease) or (2) an estimated ten-year CVD risk ≥30% or (3) an estimated risk≥20% and a systolic blood pressure (BP) ≥ 140mmHg. Risk estimates are based on World Health Organisation/ International Society for Hypertension risk prediction charts.

Randomisation
Cluster randomization will occur at the level of the Primary Health Centre (PHC) with 3 villages per PHC participating. Following an initial 6 month control phase, six PHCs will be randomised to the intervention over three time intervals or 'steps' of 6 months duration (18 PHCs and 54 villages in total, 24 months duration) according to the following table:

	**Time interval**			
**Number**	Month 0-6	Month 7-12	Month 13-18	Month 19-24
6 PHCs (18 villages)	CONTROL	INTERVENTION	INTERVENTION	INTERVENTION
6 PHCs (18 villages)	CONTROL	CONTROL	INTERVENTION	INTERVENTION
6 PHCs (18 villages)	CONTROL	CONTROL	CONTROL	INTERVENTION

Central computer-based blinded randomisation will be done at the George Institute in Hyderabad. Allocation will be stratified by geographic region, population size and distance from a large town

Intervention
The intervention will comprise the following features:

- Equipment for ASHAs and PHC doctors to assess CVD risk using the CDSS application in a 7 inch Tablet device. A back pack sized kit, containing smart tablet, BP monitor, glucometer and other management resources will be provided.
- A shared electronic record to capture patient information via smart tablet and securely send data to a centralised server.
- A referral system to the PHC for patients identified at high CVD risk.

- A prompt system to alert high risk individuals for follow-up visits with ASHA / PHC doctor and reminders on medication adherence
- Training and resource support for ASHAs and PHC doctors

Comparator
Usual health care without access to the features described above

Data collection
Data collection will occur on 5 occasions for each village – at baseline, at each interim time-interval (i.e. each "step", and at the end of follow-up. At baseline, a complete household survey (average ~1000 households per village) will be done in each village for all consenting adults ≥40 years (average ~1300 people per village). At each subsequent time point data will be collected from a random independent sample of 15% of people at high risk (average ~50 people per village).

Quantitative Outcomes

Primary:
- Difference in proportion of high risk individuals (with or without CVD) who are achieving optimal BP levels (Systolic BP<140 mmHg) between the intervention and control periods

Secondary:
- mean reduction in BP levels,
- receipt of lifestyle advice by a health care provider,
- change in other CVD risk factors, including body mass index; waist circumference; current smoking; reported healthy eating habits; reported physical activity levels, Total/HDL cholesterol;
- self-reported use of BP and other cardiovascular medicines;
- Quality of life (using the EQ-5D)
- CVD events

Statistical power
18 PHCs (54 villages)progressively randomised by a third to the intervention will provide >90% power (2α=0.05) to detect an absolute difference of 6% in the proportion of people with optimal BP levels (defined as a systolic BP<140 mmHg). This translates to an increase in the proportion achieving optimal BP levels from 39% (based on our APRHI data) to 45% and a mean systolic BP difference of around 3 mmHg. These calculations assume an intraclass correlation coefficient (ICC) of 0.03 and 5 time-points for data collection. A cluster size of 150 patients (~50 per village) is anticipated on the basis of 23% of the adult population≥40 years being identified as high risk (~300 per village)

Economic and Process evaluation
The economic evaluation will have a trial-based component and a modeled evaluation of long term costs and outcomes. The qualitative process evaluation will involve semi-structure interviews with a diversity sample of consenting patients and staff.

Significance
This project will comprehensively explore the challenges of implementing well-established evidence into practice. The findings are likely to advance locally relevant knowledge on scaling up a strategy to overcome entrenched inequities in access to effective health care for under-served populations. By placing this research at the intersection of policy, health care providers and consumers, the evidence generated has substantial potential to inform decision-making for system planners. Such approaches, if found to be effective and cost-effective and combined with effective population-based strategies, have the potential to positively impact on the healthcare of millions of Indians on a daily basis and will have wider applicability for other LMICs.

3 STUDY SCHEMA

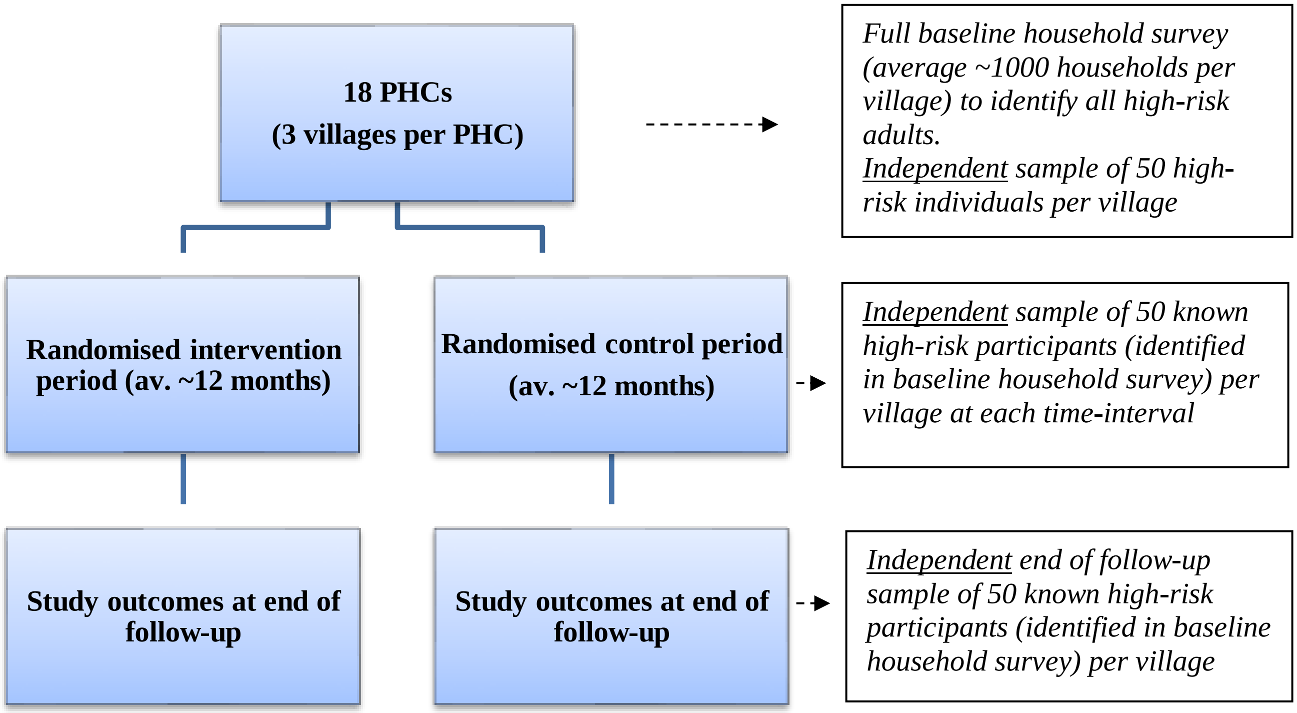

4 BACKGROUND

4.1 CVD burden in India

In India, the number of years of life lost because of coronary heart disease deaths before the age of 60 years will increase from 7·1 million in 2004 to 17·9 million in 2030, more life years lost than is projected for China, Russia, and the USA combined.[1] Health-care expenditure on chronic diseases was 70% of the average monthly income for people in low-income groups and was 45% for those in the highest income group. Elevated blood pressure is a major contributor to the increasing burden of cardiovascular disease (CVD) in India, causing almost a million deaths annually.[2] India had an estimated 118 million people diagnosed with hypertension in 2000 with projections indicating a doubling to 213 million by 2025.[3] The Indian Council of Medical Research estimates that 16% of ischaemic heart disease, 21% of peripheral vascular disease, 24% of acute myocardial infarctions and 29% of strokes in India could be attributable to hypertension.[4] This disease burden is not confined to urban India. In rural areas, where 70% of the country's population resides, high levels of hypertension and other CVD risk factors exist, with CVD already the leading cause of adult death in many such communities.[5 6]

4.2 Evidence-practice gaps in CVD prevention

Use of BP lowering treatments in rural India is limited, even where mandated low cost antihypertensive drugs are available in government formularies. We and others have shown that few people with hypertension and/or CVD are appropriately managed in these settings.[7 8] The George Institute India has collected extensive data relating to CVD and CVD risk factors in rural Andhra Pradesh,[5 7 9 10] finding that 27.0% of adults aged ≥ 30 years had hypertension. Importantly, prevalence rates were similar for men and women and around one-half were previously unidentified. In these communities, 7.7% of adults had established CVD and a further 8.1% were at high risk (defined as estimated 10-year CVD risk ≥ 30% using World Health Organisation (WHO)/ International Society of Hypertension (ISH) risk charts). For those with CVD, 25% had non-optimal BP levels (systolic BP>140mmHg). For those at high risk without known CVD, 95% had non-optimal BP levels. These rates were similarly experienced by men and women. Overall only 39% of all high risk individuals with or without CVD have adequate BP control, indicating large evidence-practice gaps and ineffective current approaches to reducing BP-related risk. In the context of limited resources, prioritising high-risk patients for BP lowering treatment is likely to be a highly cost-efficient approach and is consistent with new national primary care guidelines in India.

4.3 Workforce challenges

India's health system faces great challenges in tackling this rising CVD-related burden. Key issues include lack of health care facilities, limited access to health care providers, and high out of pocket costs for consumers.[11] It is therefore imperative that innovative solutions are developed to address these issues. India's three tier health care system, provides nurse level primary health care at the sub-centre (population ~3000-5000), doctor level care at the Primary Health Centre (PHC) (population ~ 20,000-30,000) and specialised care at the Community Healthcare Centres (population ~ 80,000-120,000). The PHC, which is usually led by one doctor, is expected to provide comprehensive primary health care for up to 30,000 residents. This leads to massive unmet demand, placing considerable strain on PHC resources and consequently quality of care provided. In this context, there is an urgent need for different workforce strategies. One promising solution is to expand the capacity of non-physician health workers with lower levels of health training. Within each village, one or more local female residents are appointed as Accredited Social Health Activists (ASHAs). ASHAs are non-physician healthcare workers with an average of ~10 years of formal education. They are selected for this role by the village Panchayat (a village based self-governance system) and receive 3 weeks of induction medical training over a 12 month period followed by on the job support. ASHAs receive performance-based remuneration under India's National Rural Health Mission programme. On average, they work for 2-3 hours each day, with a primary focus on maternal and child health. Their services are primarily provided through outreach village household visits, which provide an ideal environment for additional opportunistic health screening activities and can limit inequities arising from gender differences in healthcare seeking behaviour. A

cluster randomised controlled trial (c-RCT) of a cardiovascular risk screening strategy involving 44 villages in the region [12]. This trial demonstrated that a simple algorithm administered in the community by non-physician health workers increased the detection of CVD.[13] This suggests that this workforce can be trained to effectively identify people at high risk and refer them appropriately for medical care.

4.4 Clinical Decision Support Systems (CDSS)

Several systematic reviews have consistently shown that CDSS are able to improve effectiveness of care. In five systematic reviews on the effectiveness of CDSS, around two-thirds of controlled studies have demonstrated improvement in health care performance.[14-19] Key factors associated with improved outcomes include incorporation into usual work flow, provision of support at the time and location when care is provided, use of computer-based tools and specific advice rather than simply assessments.[16] The vast majority of high quality evaluations of CDSS have been conducted in high income countries and have targeted physicians and other health care workers with high levels of training. Their external validity in LMIC settings is unclear.[20]

4.5 Mobile-health interventions

Given its increasing ubiquity, the mobile devices (mobile phones, smartphones, tablets) represent one of the few hardware products available with the potential to increase access to health care and health information on a large scale. By providing a ubiquitous, accessible and affordable platform, there is potential for these devices to provide the essential platform for transforming the delivery of health care. Research evidence to demonstrate this, however, is still relatively immature. Despite the promise of an m-health revolution in LMICs, a recent comprehensive review concluded that the current evidence for their effectiveness is fragmented, immature and focused on intermediary outcomes such as cost savings and improved data quality.[21] Critically the review concluded that '*end-to-end patient care systems and point-of-care support for health workers are needed whereby m-health applications are interoperable and integrated with provider systems- linking the most remote community health worker with the most appropriate sources of information when and where it is needed.'p.5* [21]

5 SMARTHEALTH DEVELOPMENT

SMARTHealth is a novel electronic decision support system to facilitate guidelines-based assessment and management of CVD risk. Drawing on past experience with CDSS in the Australian primary health care setting, SMARTHealth India has been designed for use in resource-limited settings in rural India by non-physician primary health care workers and doctors. Outline below are the key development steps we have undertaken

5.1 Decision support algorithm development

A single screening and management algorithm has been developed for SMARTHealth based on a synthesis of recommendations from Indian and international screening and management guidelines. For assessment of risk WHO/ISH risk charts are used to calculate a person's 10 year absolute CVD risk. These charts are tailored to each WHO region and use age, sex, smoking status, diabetes status, systolic blood pressure to determine risk. Depending on the availability of cholesterol information, low and high information charts are provided. The SMARTHealth algorithm was programmed to incorporate these charts to predict risk and automatically defaults to the appropriate chart depending on whether cholesterol data is available. For management recommendation the National Programme for Prevention and Control of Cancer, Diabetes Cardiovascular and Stroke (NPCDCS) guidelines were used and programmed into SMARTHealth.[22]

The algorithm and user interface was then programmed for use as an application on a 7-inch smart tablet using Android 4.1 operating system. Both English and local language versions (Telugu) were developed. The algorithm was then validated using a three-level process following methods used in previous work.[23]

- **Level 1** was an iterative process where each of the variables in the algorithm was tested using deidentified data from 200 patients involved in the Andhra Pradesh Rural Health Initiative. Programming modifications were made where necessary and all variables were re-tested to ensure they were programmed correctly.
- **Level 2** testing involved independently programming the algorithm into a statistical software package. Using data from 1000 patients from a larger primary health care dataset of cardiovascular risk information, we then assessed whether the outputs from the SMARTHealth system correlated with those generated from the independently programmed version. A number of minor programming errors were identified via this process and were rectified and the algorithm was re-validated until perfect correlation was achieved.
- **Level 3** testing involved User Acceptance Testing (UAT) and field testing of the system in the setting proposed. This involved implementing the system in 11 villages for 11 ASHAs and 3 PHC doctors. Feedback on the utility of the system and suggested changes for the design interface was obtained. Detailed analyses of the pilot phase will be submitted for publication in early 2013.

5.2 Shared electronic health record

SMARTHealth allows health workers to collect consented patient information for screening and healthcare purposes via a smart tablet using the Android platform. The application then uploads this information for a doctor to review using OpenMRS- a secure, community-developed, open source, electronic medical record system platform. This will enable both ASHAs and PHC doctors to contribute to the record. ASHAs can make electronic referrals to the PHC doctor and doctors can notify the health worker via his/her tablet of the diagnosis and management plan.

6 STUDY OBJECTIVES

The SMARTHealth India study will test whether the SMARTHealth system will assist health professionals and patients in making evidence based management decisions to help prevent heart attack, stroke and related conditions.

Hypothesis: Compared to usual practice, a primary healthcare worker (ASHA) led clinical decision support system will increase the proportion of high risk individuals achieving optimal BP levels.

7 STUDY DESIGN

The intervention will be evaluated using a stepped-wedge cluster randomized, controlled trial (RCT) of two years duration.

7.1 Study population

18 PHCs (3 villages per PHC) in West Godavari District, Andhra Pradesh will participate (see statistical considerations below). The mean village population is ~4000, with one-third aged ≥40 years.

Patients will be eligible to participate if they are age≥40 years, classified at high CVD risk and indicated for blood pressuring lowering treatment based on WHO and NPCDCS guidelines. High risk is defined as the presence of the following:

- Past history of CVD
- 10-year CVD risk ≥ 30%
- 10-year CVD risk of 20-29% and a Systolic BP>140 mmHg

Risk will be calculated using the 'low information' WHO/ISH Risk algorithms for India.

Based on APRHI data [10], ~23% of adults ≥40 years (~300 individuals/ village) are likely to be classified as being at high risk with around 35% of these having established CVD.

7.2 Randomisation

Cluster randomisation will occur at the level of the PHC. Eighteen PHCs from West Godavari District in Andhra Pradesh will be selected. All PHCs must have at least one doctor regularly providing services and all doctors must be willing to participate in the study. From the region serviced by each PHC, three villages will be randomly selected (54 villages in total). Six PHCs (18 villages) will be randomised to the intervention over three time intervals or steps (Table 1). Central computer-based blinded randomisation will be done at the George Institute in Hyderabad.

Table 1. Stepped-wedge randomisation

	**Time interval**			
**Number**	Month 0-6	Month 7-12	Month 13-18	Month 19-24
6 PHCs (18 villages)	CONTROL	INTERVENTION	INTERVENTION	INTERVENTION
6 PHCs (18 villages)	CONTROL	CONTROL	INTERVENTION	INTERVENTION
6 PHCs (18 villages)	CONTROL	CONTROL	CONTROL	INTERVENTION

Our experience in working closely with villages in the region has shown that engagement in the project will be maximised if all sites are guaranteed to receive the intervention for some period of time. A stepped-wedge design will ensure that every participating PHC and village receives the intervention (for at least 6 months and an average of 12 months), while still allowing an unbiased evaluation of the effectiveness of the intervention compared to usual care. In addition, a stepped wedge design increases study power (see statistical considerations below).

7.3 Intervention arm

Figure 1 outlines the workflow and the key elements of the intervention package. The intervention comprises of the following:

- Equipment for ASHAs and PHC doctors to assess CVD risk using the CDSS application in a 7 inch Smart Tablet. A back pack sized kit, containing smart tablet, BP monitor, glucometer and other management resources will be provided.
- The application will also support ASHAs to promote lifestyle advice for the determinants of high blood pressure and CVD, in particular physical activity, healthy diet and avoidance of tobacco and alcohol.
- A shared electronic record functionality using Open MRS and Sana to capture patient information via smart tablet and securely send data to a centralised server.
- The doctors will use the electronic data transmitted by the ASHAs and interpret the decision support output for management of BP and other CVD risk factors. The decision support is based on current Indian national guidelines and will provide recommendations for BP lowering, lipid lowering or anti-platelet medications. The doctors will be advised to prescribe medications from these drug classes that are available on the essential medicine list in primary health care facilities.
- A prompt system to alert high risk individuals for follow-up visits with ASHA / PHC doctor and reminders on medication adherence.

Figure 1. Workflow and elements of the intervention package

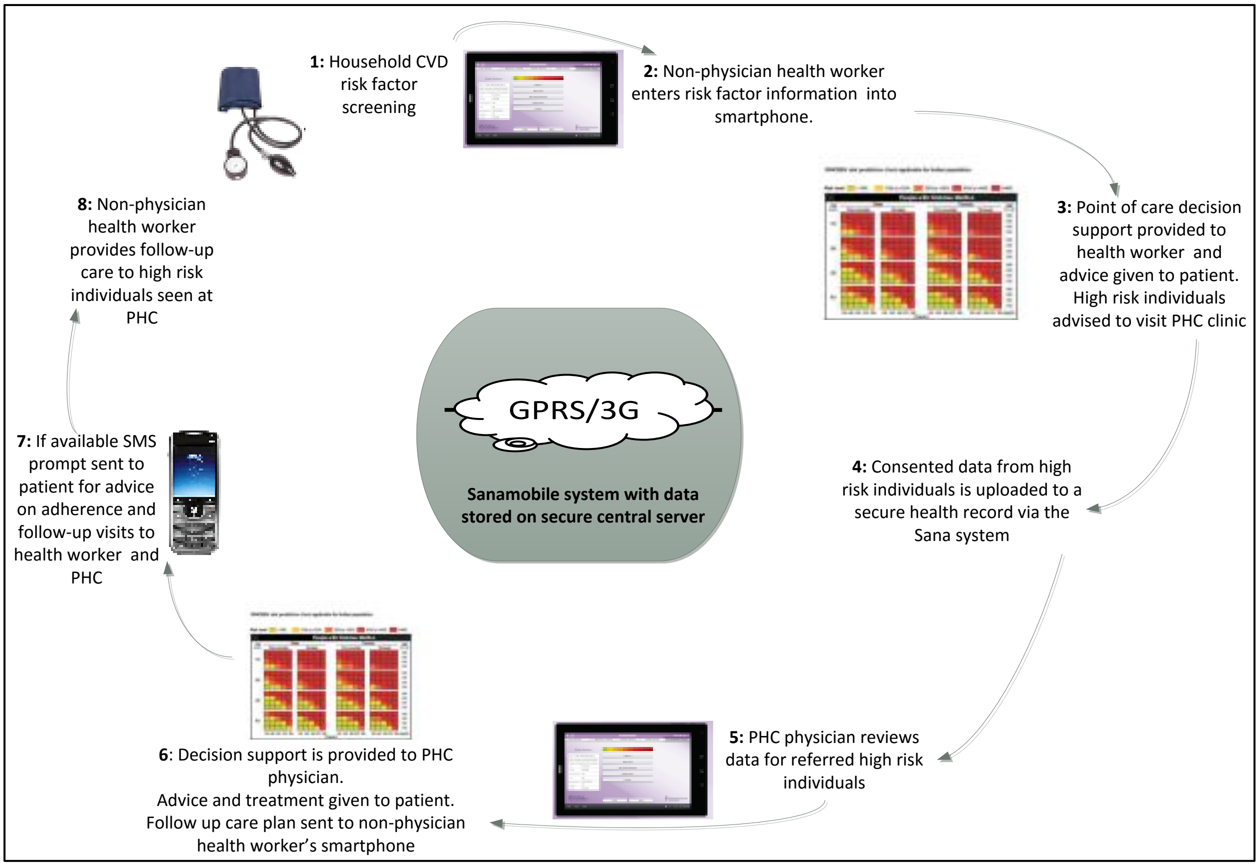

Training and resource support for ASHAs and PHC doctors- This will involve employing ASHAs on a part-time basis. ASHA training will be provided to promote awareness of lifestyle determinants of high blood pressure; use of the smart-phone CDSS to measure and record BP and other CVD risk factors; guidance in interpretation of the decision support output; provision of processes to refer high risk individuals via the SMARTHealth system to the PHC; and training to monitor and promote adherence to prescribed medications in these individuals. PHC physician training will involve guidance in the use of the electronic data transmitted by the ASHA, interpretation of the decision support output for management of BP and other cardiovascular risk factors, and advice on how to access and use audit and feedback activities. We will also explore in collaboration with the Ministry of Health strategies to enhance the current CVD medication supply to PHCs. Enhancing capacity for both the ASHA and the PHC doctor reflects a complementary approach to the proposed resource investment in the government NPCDCS program. Accordingly, remuneration to specifically implement this strategy will be a component of the intervention. Both ASHAs and physicians have a permitted private practice allocation and this is actively encouraged by district medical health officers. This will ensure study participation will not divert the ASHA or the PHC doctor from their usual duties addressing other health priorities for the population

7.4 Control arm

During control periods, access to health care will continue as per usual practice without the ASHA and PHC doctor having access to the CDSS, associated tools and the training and support package.

7.5 Data collection

The George Institute India has been actively engaged with over 50 villages in this region continuously since 2002, successfully implementing rigorous methods for data collection.[5 7 9 10 13] Data collection will occur on 5 occasions for each village – at baseline, at each interim time-interval (i.e. each "step", see table

1), and at the end of follow-up. This allows unbiased evaluation of effectiveness through comparison of "control periods" (for each village, the period between baseline and pre-intervention) and "intervention periods" (for each village, the period between pre-intervention and end of follow-up).
At baseline, a complete household survey (average ~1000 households per village) will be done in each village. As with our previous work in the region, trained field researchers will conduct interviews and make physical measurements.[5] In every household every consenting adult aged ≥40 years will be identified. Those at high risk of CVD (see 6.1) will be identified, resulting in a census of all such individuals. Any individuals with extreme elevations of blood pressure or blood sugar will be referred immediately to the PHC for treatment. At each subsequent time point data will be collected from a random independent sample of 15% of people at high risk (average ~50 people per village). This will entail administration of more detailed questionnaires, further BP measurements, anthropometry and random capillary blood glucose testing. If additional resources permit dry blood spot testing for measurement of HbA1c and lipids may also be performed. The study schema is outlined in Figure 2. It is important to emphasize that there are two independent datasets for this project: (1) the household surveys and subsequent data collection from high risk individuals and (2) the clinical data entered by ASHAs and PHC staff in the Sana system as a result of the intervention. ASHA and PHC staff will not access the evaluation data and research staff will only access deideintifed extracts of the clinical data (see ethics section below).

7.6 Primary Outcomes

- Difference in proportion of high risk individuals (with or without CVD) who are achieving optimal BP levels (Systolic BP<140 mmHg) between the intervention and control periods

7.7 Secondary Outcomes

- mean reduction in BP levels,
- change in other CVD risk factors, including body mass index; current smoking; reported physical activity levels,
- self-reported use of BP and other cardiovascular medicines;
- Quality of life (using the EQ-5D)

7.8 Statistical Considerations

18 PHC clusters (54 villages) progressively randomised by a third to the intervention (Table 1) will provide >90% power ($2\alpha=0.05$) to detect an absolute difference of 6% in the proportion of people with optimal BP levels (defined as a systolic BP<140 mmHg). This translates to an increase in the proportion achieving optimal BP levels from 39% (based on our APRHI data) to 45% and a mean systolic BP difference of around 3 mmHg. These calculations maximise the study power afforded by a step-wedge design and assume an intraclass correlation coefficient (ICC) of 0.03 (more conservative than the ICC of 0.01 previously observed in this population) and 5 time-points for data collection. A PHC cluster size of 150 patients (~50 per village) is anticipated on the basis of 23% of the adult population≥40 years being identified as high risk (~300 per village) [10], and a conservative participation rate at each survey. Methods as described by Hussey and Hughes will be used to analyse intervention effectiveness on primary and secondary outcomes, accounting for outcome variable type, clustering effects and stepped wedge design.[24]

7.9 Economic evaluation

This will have a trial-based component and a modeled evaluation of long-term costs and outcomes. The intervention cost will be based on salaries, training and other costs (smart tablets and equipment, medications, blood tests and health service visits) incurred with implementation of the intervention. Trial-based data, however, cannot capture costs and outcomes beyond the trial. A decision-analytic model will enable long-term cardiovascular morbidity, quality of life and survival to be simulated. Cost-effectiveness will be calculated in terms of incremental cost per Quality Adjusted Life Years gained. This will better inform policy makers as to the resource consequences of rolling out this program to scale.

7.10 Process evaluation

A detailed awareness of local contextual factors will be critical to understanding the impact of the intervention and any barriers to its implementation. The process evaluation will be informed by behavior change theory,[25] assessing how well the new system of service provision fits within the usual processes of current service provision in the villages and PHC centres. This will allow a better appreciation of factors that might influence sustainability beyond the trial setting. We will use mixed methods to investigate why the SMARTHealth strategy may or may not have been effective and which intervention components were most influential. Three data sources will be used: (1) de-identified usage data extracted directly from the OpenMRS system; (2) patient and provider surveys of satisfaction, tool utility and health actions taken; (3) semi-structured interviews with participants and care providers toward the end of study. For the surveys and interviews a maximum variation sampling technique will ensure diverse opinions are gained from participants, healthcare workers and district administrators. Key issues to explore include (1) how ASHAs use the intervention; (2) what effects it has on doctor practices; and (3) what are patient experiences of receiving the intervention. Data will be collected both during and at the end of the intervention period through the use of semi-structured, in-depth interviews. A number of themes will be explored including healthcare worker and consumer barriers and enablers; communication between healthcare workers and consumers; and consumer interpretation of the utility and acceptability of the intervention. Data will be analysed contemporaneously by the interview team and interview schedules will be subsequently refined as key themes emerge.

8 ETHICAL CONSIDERATIONS

The study will be conducted in accordance with the principles set out in the Declaration of Helsinki and its subsequent amendments of Tokyo, Venice, Hong Kong and Somerset West and the National Statement on Ethical Conduct in Human Research (2007). An application requesting approval to conduct this study will be submitted to the Centre for Chronic Disease Control Institutional Ethics Committee (IEC) and the University of Sydney Human Research Ethics Committee (HREC) and registered in a publically available trial registry, including compulsory registration in the Clinical Trials Registry of India. No formal review processes exist in the villages but the study and the intervention will be discussed with each village Panchayat. Informed consent will be obtained from all participants contributing data. All data collection and reporting will be compliant with national privacy law and no report will allow an individual participant to be identified. The two datasets that are generated from this project (household survey evaluation data and clinical data in the electronic health record system) will be securely stored on separate local servers at the George Institute Hyderabad. Data extracted will be deidentified and linked by a unique identification number. As required, all raw data and any derived datasets will be preserved for a period of at least 2 years from the completion of the study. All major findings will be published in peer-reviewed journals, and as policy or practice briefs to relevant stakeholders

The Principal Investigator will be responsible for producing regular status reports, serious adverse event reports, and any other required documentation to the relevant IEC/HREC in accordance with their guidelines. Any amendments or additions to the study protocol and material will be notified to the IEC/HREC by the Principal Investigator. It is the responsibility of the Principal Investigator to maintain up to date records of all correspondence and applicable documentation with the relevant IEC/HREC and the regulatory authorities. The template of the Informed Consent Forms and Patient Information Statements, together with a copy of all signed Informed Consent Forms and any other consent related correspondence will also be kept in a separate file for audit purposes. All study records and documents will be stored for a minimum of 7 years from the end of the study or for a period as required by any individual HREC.

9 TIMELINES

Task	Commencement	Completion
Ethics approval	Jan 2013	March 2013
Health Ministry Screening Committee approval (India)	Jan 2013	June 2013

Staff recruitment/ cRCT set-up	Jan 2013	June 2013
Household baseline survey in all villages	July 2013	November 2013
Randomisation and follow-up surveys of high risk individuals	December 2013	December 2015
Unblinding and analysis	January 2016	February 2016
Dissemination of findings	January 2016	ongoing

10 SIGNIFICANCE

This project focuses on a "real life" implementation of a complex intervention. It represents a case study into "Integrated Innovation"[26] incorporating a science/technology component (smart tablet, CDSS and cutting edge trial design), a social component (innovative workforce strategies) and a business component (integration with existing health system infrastructure). Despite great promise for m-health interventions to improve access to effective health care there remains considerable uncertainty about how this can be successfully achieved. These uncertainties pose substantial dilemmas for health system planners, particularly in LMICs. This project will comprehensively explore the challenges of implementing well-established evidence into practice. The findings are likely to advance locally relevant knowledge on scaling up a strategy to overcome entrenched inequities in access to effective health care for under-served populations. By placing our research at the intersection of policy, health care providers and consumers, the evidence generated has substantial potential to inform decision-making for system planners. Such approaches, if found to be effective and cost-effective and combined with effective population-based strategies, have the potential to positively impact on the healthcare of millions of Indians on a daily basis and will have wider applicability for other LMICs.

11 ADMINISTRATIVE DETAILS

11.1 Steering Committee

The Steering Committee advises on the development, implementation and evaluation of SMART Health-India (See appendix 12.1 for membership). This is a multidisciplinary team of both George Institute staff and external collaborators. In addition to its oversight of matters related to this study protocol, the Steering Committee is also responsible for overseeing new developments related to decision support, liaison with professional bodies to support the uptake of the tool in primary health care and identification of new funding opportunities and related projects.

11.2 Trial registration

The trial will be registered with the Clinical Trials Registry India, Indian Council for Medical Research and ClinicalTrials.gov

11.3 Study monitoring

This study will be monitored by a representative of The George Institute for Global Health. Site monitoring visits will be performed periodically, and communication by telephone, mail and e-mail will be used as needed to supplement site visits. The investigator and study personnel will assist the monitoring staff by providing all appropriate documentation, and being available to discuss the study.

11.4 Protocol adherence

Except for changes to eliminate an immediate hazard to participants, the approved protocol will be followed as specified. Any significant protocol deviation will be documented on a Protocol Violation Form and send to the central study management centre as soon as possible.

11.5 Protocol amendments

Any significant change in the study protocol will require an amendment. Once the study Steering Committee has approved a protocol amendment, the principal investigator will submit this to each IEC/HREC for written approval. The approval letter, signed by the IEC/HREC chair, must refer

specifically to the investigator, the protocol number, the protocol title, the protocol amendment number, and the date of the protocol amendment. The protocol amendment may be implemented only after it has been approved by the IEC/HREC. A protocol change intended to eliminate an apparent immediate hazard to subjects may be implemented immediately, but the change must then be documented in an amendment, reported to the IEC/HREC and the study Management Committee within 5 working days.

If the revision is an administrative change (such as the addition or removal of committee members), a letter explaining the change(s) along with a copy of the amended pages(s) of the protocol must be submitted to the IEC/HREC for their information. No formal approval from the IEC/HREC is required prior to implementation of administrative changes.

If any investigator retires, relocates, or otherwise withdraws from conducting a study, the responsibility for maintaining records may be transferred to The George Institute for Global Health, IEC/HREC, or other investigator. The George Institute for Global Health must be notified of and agree to the change. All associated documentation must also be updated.

11.6 Study termination

11.6.1 Termination by the Steering Committee

The Steering Committee may terminate the entire study or terminate the study at a particular centre at any time for any of the following reasons:

- Failure to enroll villages in an appropriate timeframe
- Protocol violations
- Inaccurate or incomplete data
- Unsafe or unethical practices
- Questionable safety of the intervention
- Administrative decision

11.6.2 Termination by the Investigator

If the investigator terminates the study prematurely, the investigator will do the following:

- Remove all software and hardware components related to SMART Health
- Return all study materials to The George Institute for Global Health
- Provide the IEC/HREC and The George Institute for Global Health with a written statement describing why the study was terminated prematurely

All terminations from the study will be reviewed by the Steering Committee.

11.6.3 Notification of Study Closure

In addition to interim reports as required by the IEC/HREC, the Principal Investigator will complete a final report notifying each IEC/HREC of the conclusion of the clinical study. This report should be made within 3 months of completion or termination of the study.

11.7 Records retention

The investigator shall retain and preserve one copy of all data generated in the course of the study, specifically including but not limited to those documents defined by Good Clinical Practice as essential documents, for 7 years following study closure. At the end of such period, the investigator shall notify in writing The Steering Committee of its intent to destroy all such study material. The Steering Committee shall have a further opportunity to retain such materials at The George Institute for Global Health's expense.

11.8 Indemnity

The George Institute for Global Health shall at all times indemnify the study investigators and their staff from claims that may be made against them for any injury sustained by a study participant as a consequence of effects of SMARTHealth-India in accordance with this protocol. This indemnity will be outlined in detail in the agreement between The George Institute and each participating centre.

11.9 Publication and presentations

Publication of the main report from the study will be in the name of the research group, with each individual study investigator named personally at the end of the report. All publications will be approved by the Steering Committee.

11.10 Funding

This study is funded by a National Health and Medical Research Council Global Alliances for Chronic Disease Grant (ID1040147)

12 REFERENCES

1. Patel V, Chatterji S, Chisholm D, et al. Chronic diseases and injuries in India. *The Lancet* 2011;377(9763):413-28.
2. Patel V CS, Chisholm D, Ebrahim S, Gopalakrishna G, Mathers C, Mohan V, Prabhakaran D, Ravindran RD, Reddy KS,. Chronic diseases and injuries in India. *Lancet* 2011;377:413-28.
3. Reddy KS SB, Varghese C, Ramadoss A,. Responding to the threat of chronic diseases in India *Lancet* 2005;366:1744-49.
4. Shah B KN, Menon GR, Khurana S, Kumar H, for the Indian Council of Medical Research, . Assessment of burden of non-communicable diseases.2010 Available on line at: http://www.whoindia.org/EN/Section20/Section306_1025.htm Accessed: August 2011
5. Joshi R, Cardona M, Iyengar S, et al. Chronic diseases now a leading cause of death in rural India-mortality data from the Andhra Pradesh Rural Health Initiative. *Int J Epidemiol* 2006;35(6):1522-29.
6. Kinra S BL, Lyngdoh T, et al. . Sociodemographic patterning of non-communicable disease risk factors in rural India. *BMJ* 2010;341:c4974.
7. Joshi R, Chow CK, Raju PK, et al. Fatal and Nonfatal Cardiovascular Disease and the Use of Therapies for Secondary Prevention in a Rural Region of India. *Circulation* 2009;119(DOI: 10.1161/CIRCULATIONAHA.108.819201):1950-55.
8. Jonas JB NV, Matin A, Joshi PP, Ughade SN,. Prevalence, awareness, control, and associations of arterial hypertension in a rural central India population: the Central India Eye and Medical Study. *Am J Hypertens* 2010;23:347-50.
9. Chow CK, Jolly S, Rao-Melacini P, et al. Association of diet, exercise, and smoking modification with risk of early cardiovascular events after acute coronary syndromes. *Circulation* 2010;121(6):750-8.
10. Chow CK JR, Celermajer D, et al. . Recalibration of a Framingham risk equation for a rural population in India. *J Epidemiol Community Health* 2009;63:379-85.
11. Rao M, Rao KD, Kumar AS, et al. Human resources for health in India. *The Lancet* 2011;377(9765):587-98.
12. Chow CK, Joshi R, gottumukkala AK, et al. Rationale and design of the Rural Andhra Pradesh Cardiovascular Prevention Study (RAPCAPS) - A factorial, cluster-randomised trial of two practical cardiovascular disease prevention strategies developed for rural Andhra Pradesh, India. *American Heart Journal* 2009.
13. Joshi R, Chow CK, Raju PK, et al. The Rural Andhra Pradesh Cardiovascular Prevention Study (RAPCAPS): A Cluster Randomized Trial. *Journal of the American College of Cardiology* 2012;59(13):1188-96.
14. Garg AX, Adhikari NK, McDonald H, et al. Effects of Computerized Clinical Decision Support Systems on Practitioner Performance and Patient Outcomes A Systematic Review. *Journal of the American Medical Association* 2005;293(10):1223-38.
15. Shojania KG, Jennings A, Mayhew A, et al. The effects of on-screen, point of care computer reminders on processes and outcomes of care. *Cochrane Database of Systematic Reviews: Issue 3. Art. No.: CD001096* 2009.
16. Kawamoto K, Houlihan CA, Balas AE, et al. Improving clinical practice using clinical decision support systems: a systematic review of trials to identify features critical to success. *British Medical Journal* 2005;330(7494):765.
17. Shea S, DeMouchel W, Bahamonde LA. Meta-Analysis of 16 Randomized Controlled Trials to Evaluate Computer-based Clinical Reminder Systems for Preventive Care in the Ambulatory Setting. *J. Am. Med. Inform. Assoc* 1996;3(6):399-409.
18. Souza NM SR, Mackay JA, Prorok JC, Weise-Kelly L, Navarro T,, Wilczynski NL HR. Computerized clinical decision support systems for primary preventive care: A decision-maker-researcher partnership systematic review of effects on process of care and patient outcomes. *Implement Sci* 2011 6(1):87 [Epub ahead of print] PubMed PMID: 21824381.

19. Bright TJ, Wong A, Dhurjati R, et al. Effect of Clinical Decision-Support SystemsA Systematic Review. *Ann Intern Med* 2012;157(1):29-43.
20. Ali MK, Shah S, Tandon N. Review of Electronic Decision-Support Tools for Diabetes Care: A Viable Option for Low- and Middle-Income Countries. *Journal of Diabetes Science and Technology* 2011 5(3):553-70.
21. Mechael P BH, Kaonga N, Searle S, Kwan A, Goldberger A, Fu L, Ossman J. Mechael P, Batavia H, Kaonga N, Searle S, Kwan A, Goldberger A, Fu L, Ossman J. Barriers and Gaps Affecting mHealth in Low and Middle Income Countries: Policy White Paper: Center for Global Health and Economic Development, Earth Institute, Columbia University, 2010.
22. National Programme for Prevention and Control of Diabetes Cardiovascular Disease and Stroke. Guidelines on Assessment and Management of Cardiovascular Risk for Medical Officers.2008 Available on line at: http://www.whoindia.org/LinkFiles/NMH_Resources_CVD_RISK_MANAGEMENT_BOOKLET.pdf Accessed: August 2011
23. Peiris D, Joshi R, Webster R, et al. An Electronic Clinical Decision Support Tool to Assist Primary Care Providers in Cardiovascular Disease Risk Management: Development and Mixed Methods Evaluation. *Journal of Medical Internet Research* 2009;11(4):e51.
24. Hussey MA, Hughes JP. Design and analysis of stepped wedge cluster randomized trials. *Contemp Clin Trials* 2007;28:182-91.
25. Michie S, van Stralen M, West R. The behaviour change wheel: A new method for characterising and designing behaviour change interventions. *Implementation Science* 2011;6(1):42.
26. Grand Challenges Canada/ Grand Defis Canada. Integrated Innovation.September 2010 Available on line at: http://www.grandchallenges.ca/wp-content/uploads/integratedinnovation_EN.pdf Accessed: August 2011

13 APPENDIX

Steering Committee Membership

Member	Position	Professional Organisation
David Peiris (Co-Chair)	Program Head Primary Health Care Research	The George Institute for Global Health, Australia
Anushka Patel (Co-Chair)	Chief Scientist	The George Institute for Global Health, India
Stephen McMahon	Principal Director	The George Institute for Global Health
Dorairaj Prabhakaran	Executive Director	Centre for Chronic Disease Control, New Delhi
Gari D. Clifford	Director	Centre for Doctoral Training in Healthcare Innovation, University of Oxford
Pallab Maulik	Deputy Director	The George Institute for Global Health, India
Rohina Joshi	Senior Research Fellow	The George Institute for Global Health, Australia
Stephen Jan	Senior Health Economist	The George Institute for Global Health, Australia
Stephane Heritier	Head of Statistical Research	The George Institute for Global Health
Devarsetty Praveen	Senior Research Fellow	The George Institute for Global Health, India
