# Supplementary material for: SMARThealth India: A stepped-wedge, cluster randomised controlled trial of a community health worker managed mobile health intervention for people assessed at high cardiovascular disease risk in rural India
Source: PLoS One. 2019 Mar 26;14(3):e0213708. doi: 10.1371/journal.pone.0213708 (PMC6435227; doi:10.1371/journal.pone.0213708)
Supplement: S2 File — (PDF) [file pone.0213708.s004.pdf]

SMART*health* – India

Systematic Medical Assessment Referral and Treatment in rural India

A stepped-wedge cluster randomized controlled trial of primary health care mobile health system for cardiovascular risk management in rural Andhra Pradesh

Statistical Analysis Plan

Version 6.2 October 27, 2016

Qiang Li and Stephane Heritier

Approved by: David Peiris

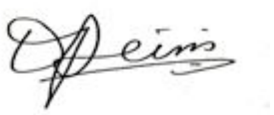

Date: 27/10/2016

Approved by:

Date:

Approved by:

Date:

Table of Contents

1. Study objective 4

2. Study Design 4

2.1 Study population 4

2.2 Intervention 4

2.3 Randomisation 5

3. Evaluation Outcomes 5

3.1 Primary outcome 5

3.2 Secondary outcome 5

3.3 Economic and Process evaluation 5

4. Data collection 6

5. Statistical analysis 6

5.1 Analysis Principle 6

5.2 Blind review 7

5.2 Baseline household survey 7

5.3 Characteristics of patients at baseline and comparison over time 7

5.4 Efficacy analyses for primary outcome 7

5.5 Analysis for secondary outcomes 9

5.6 Sub-group analyses 9

6. References 10

7. Appendix: Proposed format of data tables and figures 12

1. Study objective

The overall objective of this research is to investigate the effectiveness of an innovative and multi-disciplinary program addressing blood pressure (BP) control in rural India. The specific aims are:

a) To develop a multifaceted primary healthcare worker (ASHA) intervention that utilises a mobile device-based clinical decision support system (CDSS) to improve optimal BP control in high risk individuals.
b) To evaluate this program utilising a mixed methods evaluation in a cluster randomised trial involving villages in rural Andhra Pradesh.

2. Study design

Stepped-wedge, cluster, randomised controlled trial

2.1 Study population

Adults ≥40 years at high cardiovascular disease (CVD) risk defined as at least one of the following:

(1) past history of CVD (either coronary heart disease, stroke/ transient ischemic attack or treated peripheral vascular disease) or
(2) an estimated ten-year CVD risk ≥ 30% or
(3) an estimated ten-year CVD risk ≥ 20% and a systolic blood pressure (BP) ≥ 140mmHg or
(4) a systolic BP >= 160mmHg or
(5) a diastolic BP >=100mmHg

Risk estimates and indications for treatment are based on World Health Organization/ International Society for Hypertension risk prediction charts and Indian national guidelines

2.2 Intervention

The intervention will comprise the following features:

- Equipment for ASHAs and PHC doctors to assess CVD risk using the CDSS application in a 7-inch Tablet device. A back pack sized kit, containing smart tablet, BP monitor, glucometer and other management resources will be provided.
- A shared electronic record to capture patient information via smart tablet and securely send data to a centralised server.
- A referral system to the PHC for patients identified at high CVD risk.
- A prompt system to alert high risk individuals for follow-up visits with ASHA / PHC doctor and reminders on medication adherence
- Training and resource support for ASHAs and PHC doctors

2.3 Randomisation

Cluster randomisation will occur at the level of the Primary Health Centre (PHC) with 3 villages per PHC participating. Following an initial 6-month control phase, six PHCs will be randomised to the intervention over three time intervals or 'steps' of 6 months duration (18 PHCs and 54 villages in total, 24 months duration) according to the following table:

	**Time interval**			
**Number**	Month 0-6	Month 7-12	Month 13-18	Month 19-24
6 PHCs (18 villages)	CONTROL	INTERVENTION	INTERVENTION	INTERVENTION
6 PHCs (18 villages)	CONTROL	CONTROL	INTERVENTION	INTERVENTION
6 PHCs (18 villages)	CONTROL	CONTROL	CONTROL	INTERVENTION

Central computer-based blinded randomisation will be done at the George Institute in Hyderabad. Allocation will be stratified by geographic region, population size and distance from a large town

3. Evaluation outcomes

3.1 Primary outcome

Difference in proportion of high risk individuals (with or without CVD) who are achieving optimal BP levels (define as a systolic BP < 140 mmHg) between the intervention and control periods. At the patient level, the primary endpoint is binary (i.e. whether the optimal SBP level is achieved in a high risk patient)

3.2 Secondary outcomes

- Difference in change in mean SBP and DBP levels from baseline in the high risk population
- Difference in change in other CVD risk factors from baseline, including body mass index; current smoking; reported physical activity levels in the high risk population.
- Difference in change in self-reported use of BP and other cardiovascular medicines from baseline in the high risk population.
- Difference in quality of life (using the EQ-5D) in the high risk population.
- Difference in all cause mortality and CVD events (based on self-report and verified by verbal autopsy).

3.3 Economic and process evaluation

The economic evaluation will have a trial-based component and a modeled evaluation of long term costs and outcomes. The process evaluation will involve a mixed methods analysis of trial data, data entered into the SMART*health* system during the intervention, field reports, document analysis and semi-structured interviews with a diversity sample of consenting patients and staff.

4. Data collection

Data collection will occur on 5 occasions for each village –at baseline, at each interim time-interval (i.e. each "step", and at the end of follow-up. This allows unbiased evaluation of effectiveness through comparison of "control periods" (for each village, the period between baseline and pre-intervention) and "intervention periods" (for each village, the period between pre-intervention and end of follow-up).

At baseline, a complete household survey (average ~1000 households per village) will be done in each village. Trained field researchers will conduct interviews and make physical measurements. In every household every consenting adult aged ≥40 years will be identified. Those at high risk of CVD will be identified, resulting in a census of all such individuals. Any individuals with extreme elevations of blood pressure or blood glucose will be referred immediately to the PHC for treatment. At each subsequent time point data will be collected from a random independent sample of 15% of people at high risk (average ~50 people per village). This will entail administration of more detailed questionnaires, further BP measurements, anthropometry and random capillary blood glucose testing.

It is important to emphasize that there are two independent datasets for this project: (1) the household surveys and subsequent data collection from high risk individuals which will be used for the primary and secondary outcome evaluation and (2) the data entered by ASHAs and PHC staff in the Sana system as a result of the intervention. The latter data source will be used for the economic and process evaluations (in addition to the household data surveys). ASHA and PHC staff will not access the evaluation data and research staff will only access de-identified extracts of the clinical data.

5. Statistical analysis

5.1 Analysis principle

- A blind review will be conducted initially to assess data quality and determine the need for imputation of missing values (see below)
- Analyses will be conducted on an intention to treat basis.
- Primary analyses will be performed on the first dataset identified above only (household survey and subsequent data collection from high risk individuals).
- All statistical tests will be two-tailed. Treatment effect for the primary and secondary outcomes will be considered significant at level $\alpha=0.05$.
- We will report the number of observations used in each analysis. Summaries of continuous variables that are normally distributed will be presented as means and SDs and/or medians and inter-quartiles for skewed data, whereas categorical variables will be presented as frequencies and percentages.
- All analysis on outcomes will account for the clustering of villages and time effect.
- Analyses will be conducted primarily using SAS software.

5.2 Blind review

A blind review will be performed at baseline survey and follow-up visits to check all analysis variables. This will include assessment of missing values, frequency and percentage of categorical variables, and a descriptive summary of continuous variables and numbers of subjects used in the primary analysis. Other descriptive checks include,

1. Check the overall event rate from under-treated patients who were identified from baseline. *
2. Check the concordance and discordance of event rate between clinic data and survey data.
3. Check the overall proportion of primary events from high risk patients at each time point.
4. Check the overall CVD events at each time point

* Under-treatment of patients is defined as those people at high CVD risk who report that they are not taking any BP medications at the time of the baseline survey.

5.2 Baseline household survey

The sample size of enrolled, number high risk CVD patients will be presented in **Table 1** to present the valid patients number by PHC and villages.

5.3 Characteristics of patients at baseline and comparison over time

Description of high CVD risk patients over time will be presented in **Table 2** to present the valid number of patients in the analysis. Demographics and baseline characteristics will be presented in **Table 3** by PHCs and at each time point. The continuous variables will be presented as means and standard deviations, or medians and inter-quartile range, as appropriate. Discrete variables will be summarized by frequencies and percentages. Percentages will be calculated according to the number of patients for whom data are available. If missing values are important, the denominator will be added in.

Baseline measures for all patients will be tabulated for the following variables: age, sex, education level, occupation, marital status, history of cardiovascular diseases (prior stroke, hypertension, diabetes mellitus, and smoking status), family history (heart attack, stroke, and diabetes), blood pressure and blood glucose measurements. For the high risk cohort additional information on medical history and physical activity, quality of life and WHO well-being index will also be tabulated.

Similar patient characteristics will be presented at each time point among high CVD risk patients if the data are available.

5.4 Efficacy analyses for primary outcome

- **Descriptive results of primary outcome**

The raw percentage of high risk patients who achieved optimal BP levels will be summarized as N (%) by PHCs and time (**Table 5 and Figure 1**).

The study population used for primary outcome includes all high risk patients present in the household survey at month 6, 12, 18 and 24. Please note that the samples of high risk patients at each follow-up time period are independent.

- **Model analysis for primary outcome**

In a stepped wedge design more clusters are exposed to the intervention towards the end of the study than in its early stages (one-way). This implies that the effect of the intervention might be confounded with any underlying temporal trend. A result that initially might seem suggestive of an effect of the intervention may therefore transpire to be the result of a positive underlying temporal trend. [1]

Therefore, as a cluster randomized stepped wedge design trial, the intervention effect will be adjusted with calendar time (call "6-month interval" here) for all models. All outcomes analysis will be at the individual level, including adjustment for the clustering effect.

The primary analysis will use generalised estimating equations (GEE) with logic link function, comparing odds of percentage of achieving optimal BP level between intervention and control using all period data with time as a fixed effect, with exchangeable correlation structure to account for clustering (the correlation within PHCs). [2] As we have a small number of clusters (18 PHCs) in this study, the type 1 error of standard z-tests in the GEE model is grossly inflated due to the variance of the intervention effect estimate being too small. This problem is compounded by the necessary adjustment for time (type I error = 9-10% instead of 5% nominal).
A possible solution is provided by a small-sample adjustment to the GEE variance estimate. The Mancl De Rouen's correction[3]] available in R will be used as simulations show that that it works reasonably well for the GEE model considered here with n=18 PHCs. The 95% confidence interval (CI) for the OR will be adjusted accordingly. For the purpose of practical implementation in the statistical package SAS, the GLIMMIX procedure for the corresponding mixed model with logit link will also be used for small-sample variance correction (reference: https://www.nihcollaboratory.org/cores/Pages/Biostatistics.aspx)

The primary analysis of the primary outcome will incorporate time as a factor (class statement in SAS) and assume the same treatment effect for all time-points (see model 1 in the appendix). Since independent samples of high risk patients will be selected at the follow-up time-points, the analysis will take into account intra-class correlation at the PHC level only (rather than at patient level). A secondary analysis will also be undertaken by adjusting for potential imbalance factors at baseline; the small sample adjustment works as well if the number of covariates that are added to the model is not large (<=4). In that case the adjusted OR will reported along with the corrected 95% CI.

In addition, a sensitivity analysis will be conducted using a GEE model with time by intervention interaction – see model 2 in the appendix – to assess whether the intervention effect varies across time-points. An interaction is unlikely but this analysis will be carried out for the sake of completion. Again the small adjustment should be implemented for the interaction test(s). Two different ORs at periods 2 and 3 can be calculated as explained in the appendix. They can be presented in a figure with their 95% Cis incorporating a small sample correction similar to the one indicated above. Models equivalent to models 1 and 2 where time is introduced as a continuous covariate will be examined if deemed appropriate. Forcing a linear trend is a stringent assumption we do not want to make a priori.

Results will be presented in terms of n, % OR (95% CI) and p-value for both the adjusted and unadjusted analysis – see Table 7

The Intra-cluster coefficient (ICC) for primary outcome will be estimated from the correlation coefficient across endpoint values of patients from the same cluster. This estimate, directly provided by the GEE output, is on average slightly underestimated due to the small number of clusters but no correction will be attempted as the bias is generally small.

5.5 Analysis for secondary outcomes

For binary outcomes, we will use the similar analysis strategy as the primary outcome. For continuous outcomes, GEE models will be still used but with Gaussian link function instead of logit link function and a similar small-sample correction will be implemented. Secondary binary outcomes will be presented in separately in a format similar to the primary outcome's Tables 7 (). Gaussian endpoints will be summarized by means (SD) and the intervention effect by the overall mean difference and 95% CI following a similar structure. Again ICCs will be provided by the GEE model fits in a separate table.

5.6 Sub-group analyses

Pre-specified sub-group analyses will be conducted at the patient levels.
Patient level sub-groups include the following:

1. Attainment of the primary outcome at baseline
2. BP treatment status at baseline
3. Past history of established CVD
4. Individuals identified by ASHAs to be at high CVD risk
5. Sex
6. Use of private/public doctor (collected only in the clinical data (follow-up by ASHAs)

The analysis will be based on a GEE model similar to model 1 with the addition of the corresponding subgroup and its interaction with the intervention. The interaction test and its small-sample corrected p-value will be reported along with ORs and 95% CI within each category.

PHC level sub-groups include:

1. PHC size based on attributed population
2. PHCs with 80% or more government appointed ASHAs.
3. Availability of PHC doctors at the PHC for a minimum of 50% or more intervention time.

6. References

1. Hemming K, Haines TP, Chilton PJ, Girling AJ, Lilford RJ. *The stepped wedge cluster randomised trial: rationale, design, analysis, and reporting.* BMJ. 2015 Feb 6; 350: h391. doi: 10.1136/bmj.h391.
2. Hussey MA, Hughes JP. *Design and analysis of stepped wedge cluster randomized trials.* Contemp Clin Trials. 2007 Feb;28(2):182-91. Epub 2006 Jul 7. Review.
3. Mancl LA, De Rouen T. *A Covariance estimator for GEE with improved small-sample properties*. Biometrics 57(1):124-34

7. Appendix – Analysis models

Model 1 (Primary analysis model)
We will first fit a full rank model (**Model 1**) in which include the main effect of intervention (*I*) and calendar time as a categorical variable. The interaction between intervention and time periods is also fitted in the model to take into account any possible significant interactions, assuming the intervention effects are not constant among four periods with different starting intervention.

The probability of primary outcome in Model 1 for patient *k* in cluster *i* at time *j* can be modelled as:

$$Logit(p_{ijk}) = \beta_0 + \beta_1 {}^* x_{ij} + \beta_2 time2_{ij} + \beta_3 time3_{ij} + \beta_4 time4_{ij}$$

where x_{ij} is the intervention indicator for patient *k* in cluster *i* at time *j,* $time2_{ij}$ the time indicator for the 2nd period (Month 7-12), $time3_{ij}$ the time indicator for the 3nd period (Month 13-18), and $time4_{ij}$ the time indicator for the 4nd period (Month 19-24). The first period is the reference and is therefore not included in the model.

Model 2 (sensitivity)
In model 2 the interaction between intervention and time seen as a factor will also be tested to see if there is an additional effect of time after initiating the intervention

The probability of primary outcome in Model 2 for patient *k* in cluster *i* at time *j* can be modelled as:

$$Logit(p_{ijk}) = \beta_0 + \beta_1 {}^* x_{ij} + \beta_2 time2_{ij} + \beta_3 time3_{ij} + \beta_4 time4_{ij} +$$

$$+\beta_5 {}^* x_{ij} {}^* time3_{ij}$$

The interaction model is more complicated than usual due to the interplay between period and intervention.

In period 1 (1-6 month), nobody receives the intervention so this time point has no impact to assess its effect; in period 4 the intervention is always administered, therefore the term x_{ij}*$time4_{ij}$ is simply $time4_{ij}$ for *j*=4 so there this term is redundant and cannot be added to the model; periods 2 and 3 only contribute to compute the different ORs, only one interaction term is needed as time-point 2 (or 3) must be chosen as the reference.

Log-odds per period and intervention and ORs

	Period 1	*Period 2*	*Period 3*	*Period 4*
Control	β_0	$\beta_0 + \beta_2$	$\beta_0 + \beta_3$	NA
Intervention	NA	$\beta_0 + \beta_1 + \beta_2$	$\beta_0 + \beta_1 + \beta_3 + \beta_5$	$\beta_0 + \beta_1 + \beta_4$
OR	NA	$\exp(\beta_1)$	$\exp(\beta_1 + \beta_5)$	NA

If the interaction term is not significant, the initial (default) model is preferable

8. Appendix: Proposed format of data tables and figures

Table 1. The sample size of enrolled, completed and identified high CVD risk patients by PHC groups and villages

		Baseline household survey		
		Enrolled N	Completed N	High CVD risk N(%)
PHCs grp 1	Village 1	xxx	xxx	xxx
PHCs grp 1	Village 2	xxx	xxx	xxx
PHCs grp 1	Village 3	xxx	xxx	xxx
PHCs grp 1	…	xxx	xxx	xxx
PHCs grp 2	Village 19	xxx	xxx	xxx
PHCs grp 2	…	xxx	xxx	xxx
PHCs grp 3	Village 37	xxx	xxx	xxx
PHCs grp 3	…	xxx	xxx	xxx
In Total		xxx	xxx	xxx

*Note: Completed means having the outcome measurements.

Table 2. Eligible high CVD risk patients for primary outcome at each time point

	Baseline	Time 6	Time 12	Time 18	Time 24	In total
	Completed (n)	Completed (n)	Completed (n)	Completed (n)	Completed (n)	Completed (n)
PHCs grp 1	xxx	xxx	xxx	xxx	xxx	xxx
PHCs grp 2	xxx	xxx	xxx	xxx	xxx	xxx
PHCs grp 3	xxx	xxx	xxx	xxx	xxx	xxx
In Total	xxx	xxx	xxx	xxx	xxx	xxx

*Note: Completed means having the outcome measurements.

Table 3. Randomization of PHCs and by time.

	Time 6	Time 12	Time 18	Time 24
	Actual number of villages randomized			
PHCs grp 1		xxx	xxx	xxx
PHCs grp 2			xxx	xxx
PHCs grp 3				xxx
In Total				xxx

Table 4. The baseline characteristics

Baseline variables	Baseline (Time 0)	
	Survey (n)	High risk pt (n/%)
Age, n mean (SD)	xxx	xxx
Female, n/N (%)	xxx	xxx
BMI, n mean (SD) , kg/m2	xxx	xxx
SBP, n mean (SD), mmHg	xxx	xxx
DBP, n mean (SD), mmHg	xxx	xxx
Glucose, n mean (SD) , mmol/L	xxx	xxx
Previous History, n/N (%)	xxx	xxx
Heart attack or angina	xxx	xxx
Stroke	xxx	xxx
Diabetes	xxx	xxx
Hypertension	xxx	xxx
Peripheral vascular disease	xxx	xxx
Family history, n/N (%)	xxx	xxx
Stroke	xxx	xxx
Diabetes	xxx	xxx
Heart attack	xxx	xxx
Smoking status, n/N (%)	xxx	xxx
Never smoked	xxx	xxx
Ex-smoker	xxx	xxx
Current	xxx	xxx
Completed high school, n/N (%)	xxx	xxx
Main occupation, n/N (%)	xxx	xxx
Manual	xxx	xxx
Business	xxx	xxx
Retired	xxx	xxx
Other	xxx	xxx
ADHAR card held, n/N (%)	xxx	xxx
Medicine history, n/N (%)	xxx	xxx
BP lowering medication	xxx	xxx
Lipid lowering medication	xxx	xxx
Anti-platelet therapy	xxx	xxx
Any other western medication	xxx	xxx
Any herbal or AYUSH medicine?	xxx	xxx
Physical activity, n mean (SD)<br>Median (IQR)	xxx	xxx
inactive *	xxx	xxx
minimally active *	xxx	xxx
HEPA active *	xxx	xxx
EQ5D utility score, n mean (SD)	xxx	xxx

Median (IQR)		
WHO well-being index**	xxx	xxx
<=28		
>28 to <=50		
>50		

* Physical activity cut points: could be classified into three levels – inactive, minimally active, HEPA active (health enhancing physical activity). [3]: Guidelines for Data Processing and Analysis of the International Physical Activity Questionnaire (IPAQ) - Short Form.

**Each of the five items is rated on a 6-point Likert scale from 0 (= not present) to 5 (= constantly present). Scores are summated, with raw score ranging from 0 to 25. Then the scores are transformed to 0-100 by multiplying by 4, with higher scores meaning better well-being. Evidence suggests a score of 50 or below is indicative for low mood, though not necessarily depression. A score of 28 or below indicates likely depression and warrants further assessment (diagnostic interview) to confirm depression.

Table 5. The characteristics of high risk patients selected at each time point

Baseline variables	Time 6	Time 12			Time 18			Time 24
	control	total	Intervention	control	total	Intervention	control	intervention
Age, n mean (SD)	xxx	xxx	xxx	xxx	xxx	xxx	xxx	xxx
Female, n/N (%)	xxx	xxx	xxx	xxx	xxx	xxx	xxx	xxx
BMI, n mean (SD) , kg/m2	xxx	xxx	xxx	xxx	xxx	xxx	xxx	xxx
SBP, n mean (SD), mmHg	xxx	xxx	xxx	xxx	xxx	xxx	xxx	xxx
DBP, n mean (SD), mmHg	xxx	xxx	xxx	xxx	xxx	xxx	xxx	xxx
Glucose, n mean (SD) , mmol/L	xxx	xxx	xxx	xxx	xxx	xxx	xxx	xxx
Previous History, n/N (%)	xxx	xxx	xxx	xxx	xxx	xxx	xxx	xxx
Heart attack or angina	xxx	xxx	xxx	xxx	xxx	xxx	xxx	xxx
Stroke	xxx	xxx	xxx	xxx	xxx	xxx	xxx	xxx
Diabetes	xxx	xxx	xxx	xxx	xxx	xxx	xxx	xxx
Hypertension	xxx	xxx	xxx	xxx	xxx	xxx	xxx	xxx
Peripheral vascular disease	xxx	xxx	xxx	xxx	xxx	xxx	xxx	xxx
Family history, n/N (%)	xxx	xxx	xxx	xxx	xxx	xxx	xxx	xxx
Stroke	xxx	xxx	xxx	xxx	xxx	xxx	xxx	xxx
Diabetes	xxx	xxx	xxx	xxx	xxx	xxx	xxx	xxx
Heart attack	xxx	xxx	xxx	xxx	xxx	xxx	xxx	xxx
Smoking status, n/N (%)	xxx	xxx	xxx	xxx	xxx	xxx	xxx	xxx
Never smoked	xxx	xxx	xxx	xxx	xxx	xxx	xxx	xxx
Ex-smoker	xxx	xxx	xxx	xxx	xxx	xxx	xxx	xxx
Current	xxx	xxx	xxx	xxx	xxx	xxx	xxx	xxx
Completed high school, n/N (%)	xxx	xxx	xxx	xxx	xxx	xxx	xxx	xxx
Main occupation, n/N (%)	xxx	xxx	xxx	xxx	xxx	xxx	xxx	xxx
Manual	xxx	xxx	xxx	xxx	xxx	xxx	xxx	xxx
Business	xxx	xxx	xxx	xxx	xxx	xxx	xxx	xxx

Retired	xxx	xxx	xxx	xxx	xxx	xxx	xxx	xxx
Other	xxx	xxx	xxx	xxx	xxx	xxx	xxx	xxx
ADHAR card held, n/N (%)	xxx	xxx	xxx	xxx	xxx	xxx	xxx	xxx
Medicine history, n/N (%)	xxx	xxx	xxx	xxx	xxx	xxx	xxx	xxx
BP lowering medication	xxx	xxx	xxx	xxx	xxx	xxx	xxx	xxx
Lipid lowering medication	xxx	xxx	xxx	xxx	xxx	xxx	xxx	xxx
Anti-platelet therapy	xxx	xxx	xxx	xxx	xxx	xxx	xxx	xxx
Any other western medication	xxx	xxx	xxx	xxx	xxx	xxx	xxx	xxx
Any herbal or AYUSH medicine?	xxx	xxx	xxx	xxx	xxx	xxx	xxx	xxx
Physical activity, n mean (SD)<br>Median (IQR)	xxx	xxx	xxx	xxx	xxx	xxx	xxx	xxx
inactive *	xxx	xxx	xxx	xxx	xxx	xxx	xxx	xxx
minimally active *	xxx	xxx	xxx	xxx	xxx	xxx	xxx	xxx
HEPA active *	xxx	xxx	xxx	xxx	xxx	xxx	xxx	xxx
EQ5D utility score, n mean (SD)<br>Median (IQR)	xxx	xxx	xxx	xxx	xxx	xxx	xxx	xxx
WHO will-being index**	xxx	xxx	xxx	xxx	xxx	xxx	xxx	xxx
<=28								
>28 to <=50								
>50								

***** Physical activity cut points: could be classified into three levels – inactive, minimally active, HEPA active (health enhancing physical activity). [3]: Guidelines for Data Processing and Analysis of the International Physical Activity Questionnaire (IPAQ) - Short Form.

**Each of the five items is rated on a 6-point Likert scale from 0 (= not present) to 5 (= constantly present). Scores are summated, with raw score ranging from 0 to 25. Then the scores are transformed to 0-100 by multiplying by 4, with higher scores meaning better well-being. Evidence suggests a score of 50 or below is indicative for low mood, though not necessarily depression. A score of 28 or below indicates likely depression and warrants further assessment (diagnostic interview) to confirm depression.

Table 6. Description of primary outcome events in N (%) among high CVD risk patients by PHCs and by time.

	Time 0	Time 6	Time 12	Time 18	Time 24
	N (%)	N (%)	N (%)	N (%)	N (%)
PHCs grp 1	xxx	xxx	xxx	xxx	xxx
PHCs grp 2	xxx	xxx	xxx	xxx	xxx
PHCs grp 3	xxx	xxx	xxx	xxx	xxx
In Total	xxx	xxx	xxx	xxx	xxx

Figure 1. mean plot (illustration only):

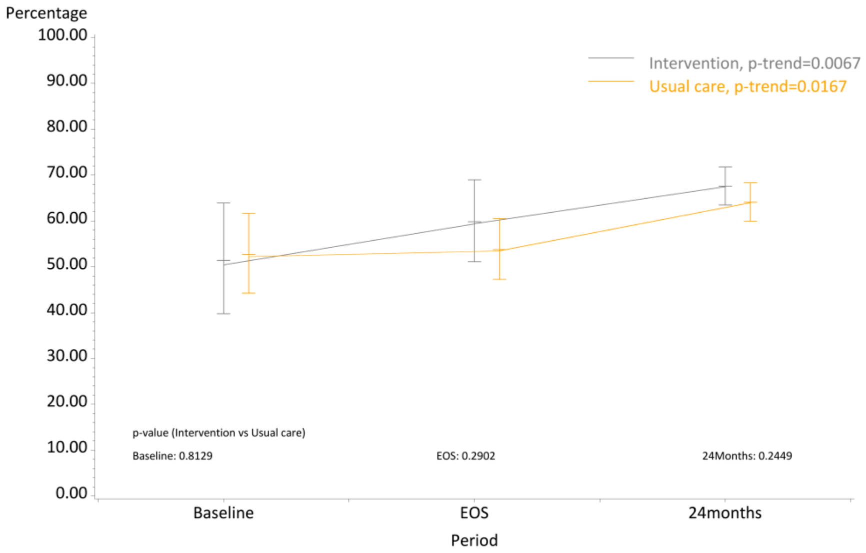

Table 7. The effect of intervention (odds ratio) with their 95%CI and p values from Model 1 for primary outcome.

	Control		Intervention		Model 1		
	**n**	**%**	**n**	**%**	**OR (95%CI)**	**P-value**	**P-value (adjusted)**
Primary outcome	xxx	xxx	xxx	xxx	xxx	xxx	xxx
Proportion of high risk patients achieved BP level (SPB<140mmHg)	xxx	xxx	xxx	xxx	xxx	xxx	xxx
Secondary outcomes							
Mean reduction in SBP and DBP levels	xxx	xxx	xxx	xxx	xxx	xxx	xxx
Change in BMI	xxx	xxx	xxx	xxx	xxx	xxx	xxx
Change in smoking status	xxx	xxx	xxx	xxx	xxx	xxx	xxx
Change in physical activity levels	xxx	xxx	xxx	xxx	xxx	xxx	xxx
Change in self-reported taking of at least one BP medicine	xxx	xxx	xxx	xxx	xxx	xxx	xxx
Change in EQ5D utility score	xxx	xxx	xxx	xxx	xxx	xxx	xxx
Incidence of all-cause fatal events	xxx	xxx	xxx	xxx	xxx	xxx	xxx
Incidence of fatal and non-fatal CVD events	xxx	xxx	xxx	xxx	xxx	xxx	xxx

Table 8. ICC for each outcome from first time interval without any intervention

	ICC
**Primary outcome**	
Proportion of high risk patients achieved BP level (SPB<140mmHg)	xxx
**Secondary outcomes**	
Mean reduction in SBP and DBP levels	xxx
Change in BMI	xxx
Change in smoking status	xxx
Change in physical activity levels	xxx
Change in self-reported taking of at least one BP medicine	xxx
Change in EQ5D utility score	xxx
Incidence of all-cause fatal events	xxx
Incidence of fatal and non-fatal CVD events	xxx
